# Senomorphic miR-3155b suppresses the senescence-associated secretory phenotype in therapy-induced senescence

**DOI:** 10.3389/fcell.2026.1868137

**Published:** 2026-09-28

**Authors:** Kunling Huang, Juan Chen, Yunying Liu, Chuyang Ding, Caiying Yi, Wenbin Ma, Su Wu, Feng Liu

**Affiliations:** 1 MOE Key Laboratory of Gene Function and Regulation, State Key Laboratory of Biocontrol, Guangzhou Key Laboratory of Healthy Aging Research, School of Life Sciences, Institute of Healthy Aging Research, Sun Yat-sen University, Guangzhou, China; 2 Sun Yat-sen Memorial Hospital, Sun Yat-sen University, Guangzhou, China

**Keywords:** luminal A breast cancer, miR-3155b, miRNAs, senescence-associated secretory phenotype, therapy-induced senescence

## Abstract

Senomorphic microRNAs (miRNAs) are a class of small regulatory RNAs that selectively suppress the senescence-associated secretory phenotype (SASP) without eliminating senescent cells, thereby offering a targeted approach to counteract the pro-tumorigenic consequences of therapy-induced senescence (TIS). Despite their therapeutic potential, senomorphic miRNAs in luminal breast cancer remain poorly characterized. Here, we performed small RNA sequencing to profile miRNA expression changes in a doxorubicin-induced TIS model using the luminal breast cancer line MCF7. We identified miR-3155b as a previously uncharacterized miRNA significantly downregulated during TIS. Functional studies revealed that miR-3155b overexpression suppressed the mRNA and protein levels of multiple SASP factors, including IL6, IL8 (CXCL8), MMP13, TNF, IL1B, and IFNB1, whereas its knockdown enhanced their expression. Critically, these effects occurred without affecting core senescence phenotypes such as proliferation arrest, SA-β-gal activity, or DNA damage foci formation. This senomorphic function was further confirmed in an ionizing radiation (IR)-induced TIS model and in T47D luminal breast cancer cells. miR-3155b was confirmed to target the *CXCL8* mRNA by luciferase reporter assays, and RNA pull-down assays indicated that miR-3155b physically associates with *IFNB1*, *IL6*, *TNF*, and *MMP13* mRNAs. Collectively, our findings identify miR-3155b as a senomorphic miRNA in Luminal A breast cancer TIS and provide a potential target for optimizing chemoradiotherapy strategies aimed at mitigating SASP-driven adverse effects.

## 1 Introduction

Breast cancer is the most frequently diagnosed cancer and a leading cause of cancer-related mortality among women globally, posing a severe threat to female health (Siegel et al., 2026; Sung et al., 2021; Bray et al., 2024). Molecularly, breast cancer is classified into Luminal A, Luminal B, HER2-enriched, and triple-negative subtypes, with luminal breast cancer accounting for over 60% of cases (Monte et al., 2026). For high-risk or locally advanced luminal breast cancer, combined chemoradiotherapy is an important treatment strategy (Burstein et al., 2025; Mamounas et al., 2025).

A central mechanism underlying chemoradiotherapy is therapy-induced senescence (TIS), wherein tumor cells undergo stable cell cycle arrest (Colucci et al., 2025; Wang et al., 2022; Schmitt et al., 2022; Chembukavu and Lindsay, 2024). Senescent cells exhibit active secretion of cytokines, chemokines, matrix metalloproteinases, and growth factors, a phenotype termed the senescence-associated secretory phenotype (SASP) (Coppé et al., 2008). In the tumor microenvironment, TIS-related SASP exerts dual roles: while TIS contributes to antitumor efficacy by halting cancer cell proliferation, SASP can paradoxically promote epithelial-mesenchymal transition, immune evasion, metastasis, and therapy resistance. Therefore, selectively inhibiting the protumorigenic aspects of SASP while preserving the antiproliferative benefits of TIS is a key question in senescence-targeted therapy (Faget et al., 2019; Cao et al., 2025).

Two main intervention strategies have emerged. Senolytics eliminate senescent cells to remove the source of SASP (Wang et al., 2019; Sieben et al., 2018), but they lack specificity, causing off-target toxicity and disrupting tissue homeostasis (Barthet and Lowe, 2025). In contrast, senomorphic strategy modulates senescent cell phenotypes to inhibit SASP without eliminating senescent cells, offering a precise alternative (Raffaele and Vinciguerra, 2022).

MicroRNAs (miRNAs), ∼22-nucleotide non-coding RNAs, post-transcriptionally regulate gene expression by binding to the 3′untranslated region or coding region of target mRNAs, which are widely involved in senescence and inflammation (Chen et al., 2025; Turko et al., 2025; Li et al., 2020; Luo et al., 2015). Thus, miRNAs are ideal senomorphic candidates, with some termed “senomorphic miRNAs” for selectively inhibiting SASP without eliminating senescent cells (Zhang et al., 2025a; Zhang et al., 2025b). However, the majority of studies on senomorphic miRNAs focus on replicative or physiological cell senescence, with few reports in TIS models (Wang et al., 2022; Zhang et al., 2025b; Chambers et al., 2021; Mchugh et al., 2025; Shen et al., 2019; Santiago et al., 2024). Specifically, no systematic study has identified miRNAs that selectively regulate SASP in TIS from luminal breast cancer without interfering with core senescence. Given that TIS exhibits distinct SASP profiles and signaling dependencies compared to physiological senescence, identifying senomorphic miRNAs that fine-tune SASP in the TIS holds unique therapeutic potential.

Using the Luminal A breast cancer cell line MCF7 as the primary model, we established TIS models via doxorubicin and ionizing radiation to examine the role of miR-3155b across different senescence-inducing contexts. Through small RNA sequencing and functional assays, we identified miR-3155b as a senomorphic miRNA that selectively suppresses the SASP without affecting core senescence hallmarks, and further validated its function in T47D cells. These findings provide a potential target to refine chemoradiotherapy by uncoupling beneficial senescence from detrimental SASP effects.

## 2 Results

### 2.1 Doxorubicin induces a robust therapy-induced senescence in MCF7 cells

To determine the optimal conditions for doxorubicin (DOXO)-induced senescence in MCF7 cells, we evaluated cell viability via the CCK-8 assay, and assessed mRNA expression levels of established senescence-associated markers, *IL6*, *CXCL8*, and *CDKN1A* (*p21*), across a range of DOXO concentrations and treatment durations. Treatment with 120 nM DOXO for 72 h resulted in significantly reduced cell viability and marked upregulation of *IL6*, *CXCL8*, and *CDKN1A* mRNA levels compared to vehicle-treated controls (Supplementary Material, Supplementary Figures S1A-F). This condition was therefore selected for all subsequent experiments.

Under these optimized conditions, cell growth curve analysis demonstrated a pronounced and sustained proliferation arrest in DOXO-treated MCF7 cells (Figure 1A). In addition, the DOXO-treated senescence group exhibited a significantly higher percentage of SA-β-gal-positive cells compared with the vehicle-treated control group (Figures 1B,C). RT-qPCR results further confirmed that the mRNA levels of the senescence-associated genes *CDKN1A*, *IL6*, and *CXCL8* were significantly upregulated in senescent cells (Figure 1D). Moreover, immunofluorescence staining showed a pronounced increase in γH2AX foci, a well-recognized marker of DNA double-strand breaks, in senescent cells (Figures 1E,F).

**FIGURE 1:**
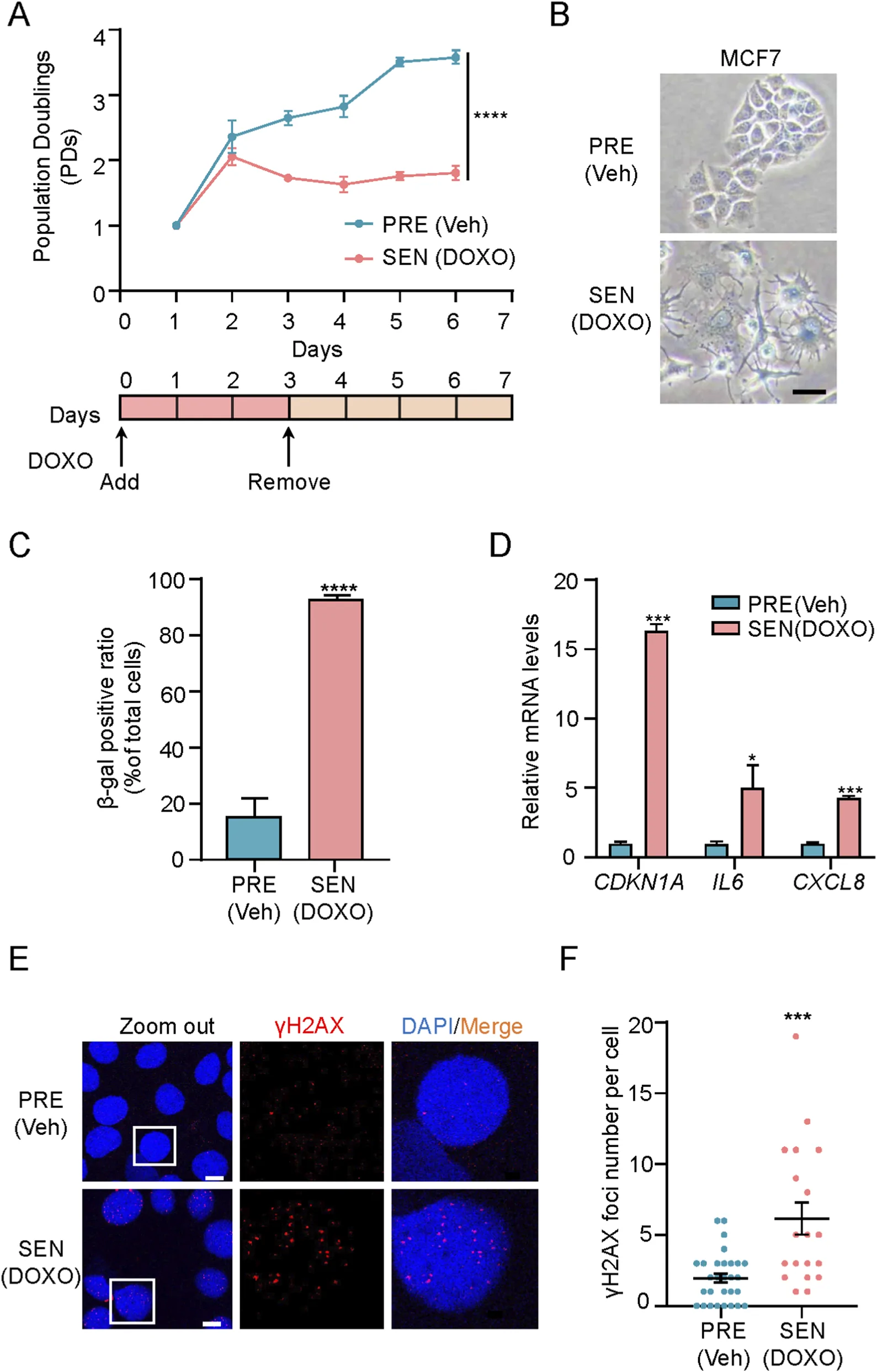
Doxorubicin induces a robust therapy-induced senescence in MCF7 cells. **(A)** Cell growth curve of MCF7 cells treated with 120 nM doxorubicin (DOXO) for 72 h, assessed by CCK-8 assay. **(B,C)** Representative images of SA-β-gal staining and quantification of SA-β-gal-positive cells in presenescent (PRE-Veh, vehicle control, DMSO) and senescent (SEN-DOXO, doxorubicin-treated) MCF7 cells. Scale bar: 10 μm **(D)** Relative mRNA expression levels of senescence-associated genes *CDKN1A*, *IL6*, and *CXCL8* in PRE (Veh) and SEN (DOXO) MCF7 cells, assessed by RT-qPCR. **(E,F)** Representative immunofluorescence images of γH2AX foci, a marker of DNA double-strand breaks, and quantification of γH2AX-positive cells in PRE (Veh) and SEN (DOXO) MCF7 cells. Scale bar: 10 μm. Data are presented as mean ± SEM from three biological replicates. For A, two-way ANOVA was used. For B–F, Student’s t-test was used. *p < 0.05, ***p < 0.001, ****p < 0.0001.

Collectively, these results indicate that a stable therapy-induced senescence model was successfully established in MCF7 cells following 120 nM DOXO treatment for 72 h.

### 2.2 Small RNA sequencing identifies downregulated miR-3155b in doxorubicin-induced senescence

To investigate the global miRNA expression changes during DOXO-induced senescence in MCF7 cells, we performed small RNA sequencing on MCF7 cells from the presenescent (PRE-Veh, vehicle control, DMSO) and senescent (SEN-DOXO, doxorubicin-treated) groups, with three biological replicates per condition (Figure 2A). Pearson correlation analysis revealed high intra-group reproducibility (Pearson’s *r* value >0.95) and clear inter-group separation (Pearson’s *r* value = 0.927-0.943), confirming data reliability (Figure 2B).

**FIGURE 2:**
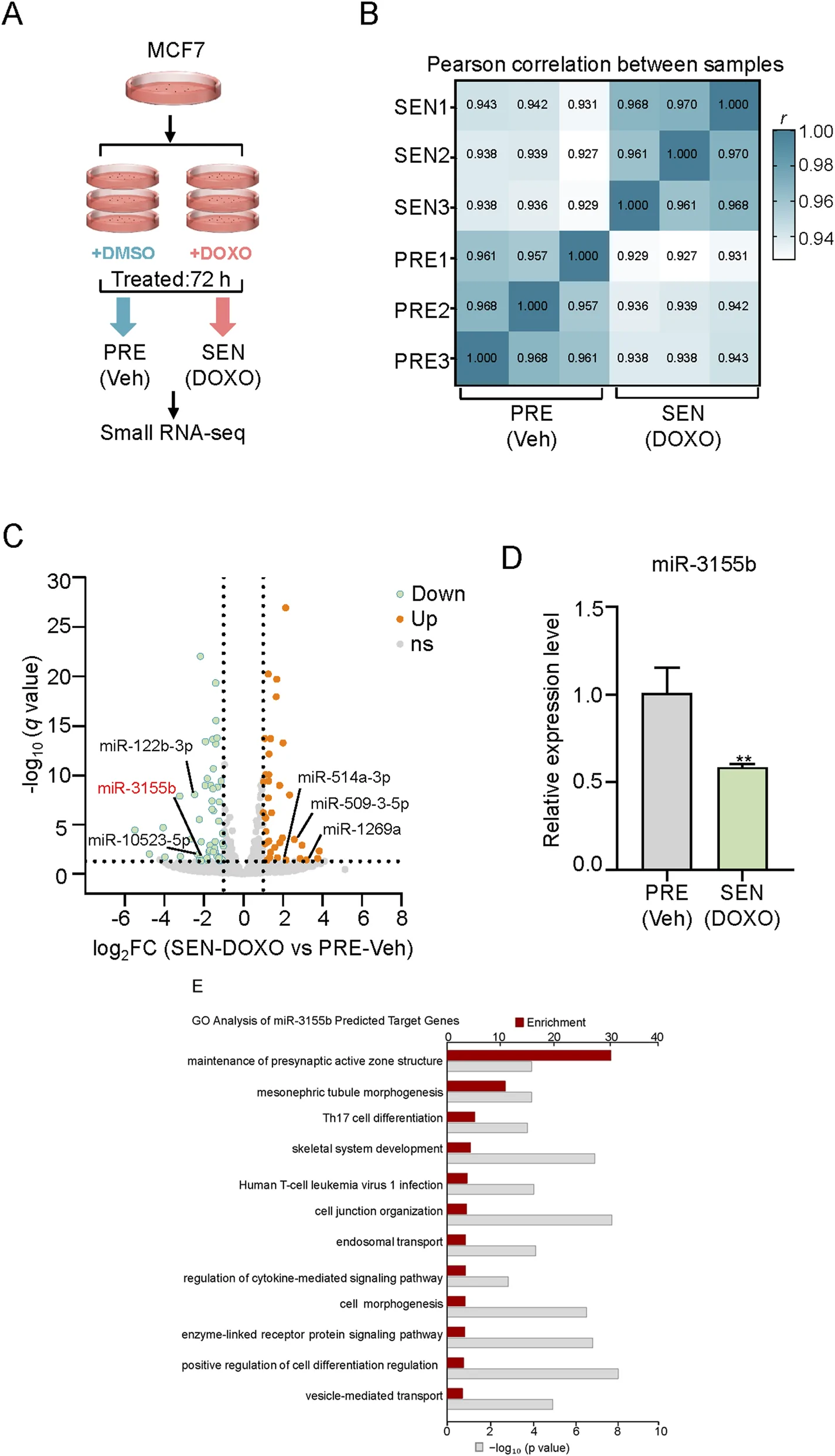
Small RNA sequencing identifies downregulated miR-3155b in doxorubicin-induced senescence. **(A)** Schematic overview of the small RNA sequencing workflow. **(B)** Heatmap showing Pearson correlation coefficients (*r*) between the six samples (three PRE-Veh and three SEN-DOXO). Color intensity represents the magnitude of correlation. **(C)** Volcano plot depicting differentially expressed miRNAs between SEN (DOXO) and PRE (Veh) groups. The x-axis represents log_2_ fold change (SEN-DOXO vs. PRE-Veh), and the y-axis represents −log_10_ (*q* value). Orange dots indicate significantly upregulated miRNAs (log_2_FC > 1, *q* < 0.05), blue dots indicate significantly downregulated miRNAs (log_2_FC < −1, *q* < 0.05), and gray dots indicate non-significant miRNAs. **(D)** Validation of miR-3155b expression levels by RT-qPCR. Data represent mean ± SEM; **p < 0.01 (Student’s t-test) **(E)** Gene Ontology (GO) enrichment analysis of predicted miR-3155b target genes. The 12 significantly enriched biological process terms are shown, ranked by enrichment score. The corresponding −log_10_ (*p* value) for each term is indicated. GO analysis was performed using Metascape with 532 predicted target genes from miRWalk (Seed = 1) as input. A total of 401 significant terms were identified. See Supplementary Table S3 for the complete list of enriched terms.

Differential expression analysis identified multiple significantly dysregulated miRNAs. The volcano plot revealed miRNAs that were significantly upregulated (log_2_FC > 1, *q* value <0.05) and significantly downregulated (log_2_FC < −1, *q* value <0.05) in the SEN (DOXO) group compared with the PRE (Veh) group (Figure 2C). To validate the sequencing results, three significantly upregulated miRNAs (miR-514a-3p, miR-509-3-5p, miR-1269a) and three significantly downregulated miRNAs (miR-122b-3p, miR-10523-5p, miR-3155b) were selected for RT-qPCR validation (Supplementary Material, Supplementary Figures S1A-E). Among these candidates, only miR-3155b exhibited consistent downregulation in senescent cells, aligning with the sequencing data (Figure 2D). Further analysis revealed that the non-validated candidates had either low expression levels or poor primer specificity. In contrast, miR-3155b showed robust read counts, significant log_2_FC (<−1), and low *q* value (<0.05), supporting its selection for further investigation.

To further explore the potential function of miR-3155b, Gene Ontology (GO) enrichment analysis was performed on its predicted target genes. The significantly enriched biological processes included synaptic structure homeostasis, Th17 cell differentiation, cell differentiation and morphogenesis, vesicle transport, and enzyme-linked receptor signaling (Figure 2E; Supplementary Table S3), suggesting that miR-3155b may participate in diverse biological processes including neural homeostasis, immune regulation, and cell development.

### 2.3 miR-3155b overexpression selectively suppresses doxorubicin-induced SASP

To investigate the functional role of miR-3155b in cellular senescence, MCF7 cells were transfected with miR-3155b mimics for overexpression experiments (Supplementary Material, Supplementary Figure S3A). RT-qPCR results showed that overexpression of miR-3155b significantly suppressed the mRNA expression levels of multiple SASP factors, including *IL6*, *CXCL8*, *MMP13*, *TNF*, *IL1B*, and *IFNB1* (Figures 3A–F). Notably, under conditions of miR-3155b overexpression, other classical senescence-associated markers remained unchanged. SA-β-gal staining revealed no significant difference in the percentage of senescent cells compared with the control group (Figures 3G,H). Furthermore, immunofluorescence analysis showed that expression of the proliferation marker Ki67 (Figures 3I,J) and the DNA damage markers γH2AX and 53BP1 (Figures 3K–N) was not significantly affected by miR-3155b overexpression. Consistently, *CDKN1A* mRNA levels remained unchanged (Supplementary Material, Supplementary Figure S3C), and this was further confirmed at the protein level by Western blot (Figures 3O,P).

**FIGURE 3:**
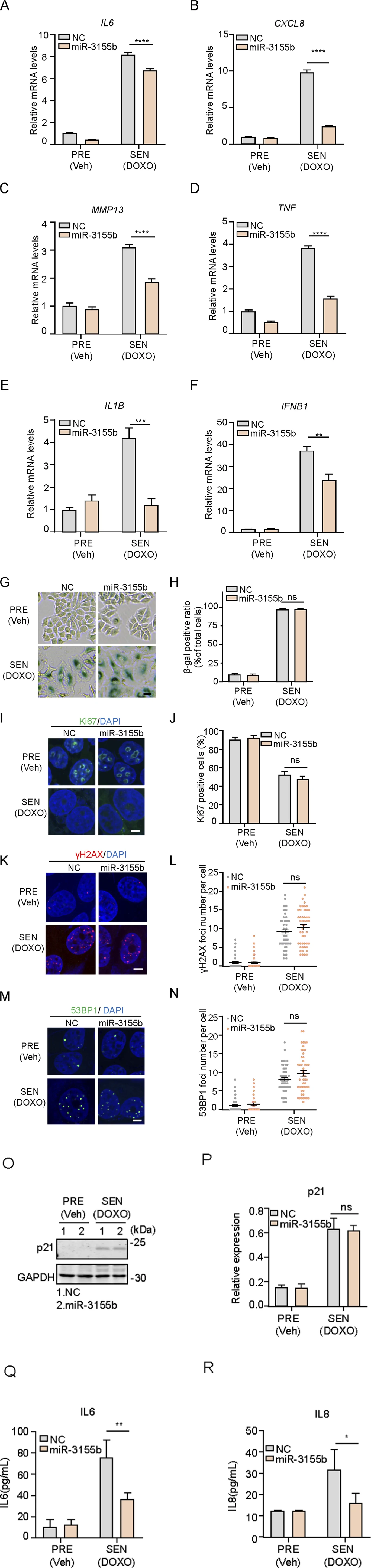
miR-3155b overexpression selectively suppresses doxorubicin-induced SASP. **(A–F)** Relative mRNA expression levels of SASP factors, including *IL6*, *CXCL8*, *MMP13*, *TNF*, *IL1B*, and *IFNB1*, in presenescent (PRE, vehicle) and senescent (SEN, DOXO) MCF7 cells, with or without miR-3155b overexpression, determined by RT-qPCR. **(G, H)** Representative images of SA-β-gal staining and quantification of SA-β-gal-positive cells. Scale bar: 20 μm ns indicates not significant. **(I, J)** Representative immunofluorescence images of Ki67 staining and quantification of Ki67-positive cells. Scale bar: 5 μm ns indicates not significant. **(K, L)** Representative immunofluorescence images of γH2AX foci and quantification of γH2AX-positive cells. Scale bar: 5 μm ns indicates not significant. **(M, N)** Representative immunofluorescence images of 53BP1 foci and quantification of 53BP1-positive cells. Scale bar: 5 μm ns indicates not significant. **(O, P)** Western blot analysis of p21 protein levels and quantification in MCF7 cells with or without miR-3155b overexpression. ns, not significant. **(Q, R)** ELISA quantification of secreted IL6 and IL8 in conditioned medium from MCF7 cells overexpressing miR-3155b or NC. Data are presented as mean ± SEM from three biological replicates and two-way ANOVA was used. *p < 0.05, **p < 0.01, ***p < 0.001, ****p < 0.0001; ns, not significant.

Having established that miR-3155b selectively suppresses SASP at the mRNA level without reversing the senescence program, we next asked whether this suppression extends to the protein level. ELISA assays on conditioned medium revealed that miR-3155b overexpression significantly reduced the secretion of both IL6 and IL8 proteins (Figures 3Q,R), confirming that the observed transcriptional changes translate into genuine alterations of the secretory phenotype.

To assess the specificity of this effect, we examined five control transcripts unrelated to SASP or senescence—*GAPDH*, *ACTB*, *RPL13A*, *TBP*, and *PGK1*—and observed no significant changes upon miR-3155b overexpression (Supplementary Material, Supplementary Figures S3E-I). These results do not suggest widespread off-target effects.

Collectively, these results indicate that overexpression of miR-3155b suppresses the SASP at both the mRNA and protein levels without affecting other classical phenotypic features of cellular senescence, suggesting a senomorphic role of miR-3155b in DOXO-induced senescence.

### 2.4 miR-3155b knockdown enhances doxorubicin-induced SASP without altering core senescence features

To further validate the functional role of miR-3155b in cellular senescence, we performed loss-of-function experiment in MCF7 cells using miR-3155b antagomir (Supplementary Material, Supplementary Figure S3B). Knockdown of miR-3155b significantly increased the mRNA expression levels of multiple SASP factors, including *IL6*, *CXCL8*, *MMP13*, *TNF*, *IL1B*, and *IFNB1* (Figures 4A–F). Consistent with the overexpression results, miR-3155b knockdown did not affect other classical senescence-associated markers. Specifically, SA-β-gal staining revealed no significant difference in the percentage of senescent cells between the miR-3155b knockdown group and the control group (Figures 4G,H). Additionally, immunofluorescence analysis showed that the expression of the proliferation marker Ki67 (Figures 4I,J) and the DNA damage markers γH2AX and 53BP1 (Figures 4K–N) was not significantly affected. Furthermore, *CDKN1A* mRNA levels remained unchanged (Supplementary Material, Supplementary Figure S3D), which was consistent with the protein level (Figures 4O,P). ELISA assays further confirmed that miR-3155b knockdown significantly increased the secretion of IL6 and IL8 proteins (Figures 4Q,R).

**FIGURE 4:**
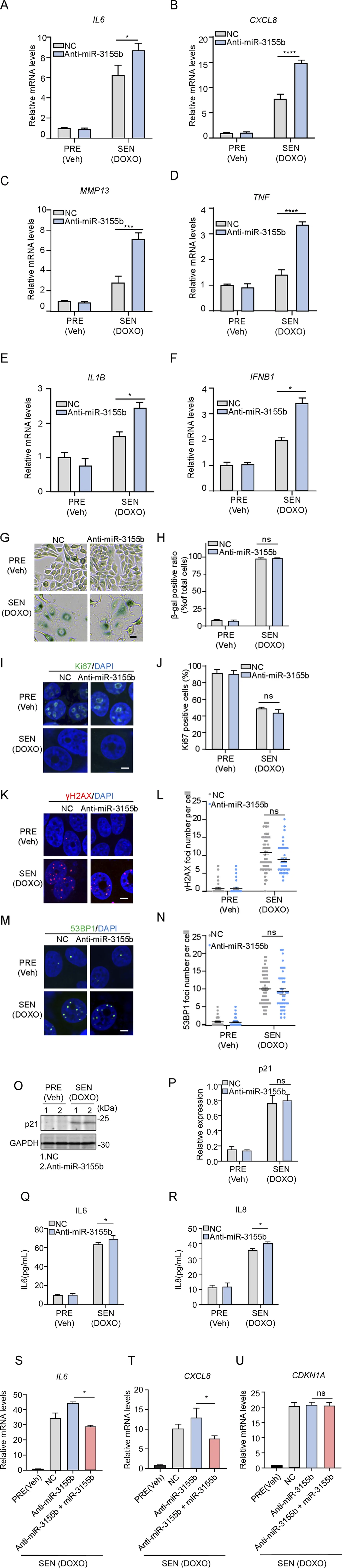
miR-3155b knockdown enhances doxorubicin-induced SASP without altering core senescence features. **(A–F)** RT-qPCR analysis of *IL6* **(A)**, *CXCL8* **(B)**, *MMP13* **(C)**, *TNF* **(D)**, *IL1B* **(E)**, and *IFNB1* **(F)** mRNA levels in DOXO-senescent MCF7 cells transfected with Anti-miR-3155b or NC antagomir. **(G,H)** Representative SA-β-gal staining images **(G)** and quantification of SA-β-gal-positive cells **(H)**. Scale bar, 20 μm. **(I,J)** Representative immunofluorescence images of Ki67 **(I)** and quantification of Ki67-positive cells **(J)**. Scale bar, 5 μm. **(K, L)** Representative immunofluorescence images of γH2AX foci **(K)** and quantification of γH2AX-positive cells **(L)**. Scale bar, 5 μm. **(M, N)** Representative immunofluorescence images of 53BP1 foci **(M)** and quantification of 53BP1-positive cells **(N)**. Scale bar, 5 μm. **(O, P)** Western blot analysis of p21 protein levels **(O)** and quantification **(P)** in DOXO-senescent MCF7 cells with or without miR-3155b knockdown. **(Q, R)** ELISA quantification of secreted IL6 **(Q)** and IL8 **(R)** in conditioned medium from DOXO-senescent MCF7 cells transfected with Anti-miR-3155b or NC antagomir. **(S–U)** Rescue experiment. RT-qPCR analysis of *CDKN1A* **(S)**, *IL6* **(T)**, and *CXCL8* **(U)** mRNA levels in DOXO-senescent MCF7 cells under the following conditions: NC antagomir + NC mimic, Anti-miR-3155b + NC mimic, and Anti-miR-3155b + miR-3155b mimic. Data are presented as mean ± SEM from three biological replicates. For A–F and S–U, two-way ANOVA with Sidak’s multiple comparisons test was used. For O, P, Q, and R, unpaired two-tailed Student’s t*-*test was used. *p < 0.05, **p < 0.01, ***p < 0.001 and ****p < 0.0001; ns, not significant.

To confirm that the SASP-regulatory effects are specifically attributable to miR-3155b, we performed rescue experiments. MCF7 cells in DOXO-induced TIS were treated with Anti-miR-3155b to neutralize endogenous miR-3155b, which led to the expected upregulation of IL6 and CXCL8 mRNA. Subsequent co-transfection with miR-3155b mimic partially reversed this elevation, reducing IL6 and CXCL8 expression. Importantly, *CDKN1A* mRNA levels remained unchanged throughout these manipulations (Figures 4S–U).

Together, these results suggest that knockdown of miR-3155b promotes the expression of SASP factors without affecting other classical phenotypic features of cellular senescence. In addition, the rescue experiment provides evidence that these regulatory effects are specifically mediated by miR-3155b, lending further support to its senomorphic role in DOXO-induced senescence.

### 2.5 miR-3155b overexpression suppresses the SASP in ionizing radiation-induced senescence

To assess whether the SASP-regulatory function of miR-3155b extends beyond DOXO-induced senescence, we established an IR-induced TIS model in MCF7 cells (Figure 5A). IR treatment (15 Gy) induced a significant increase in SA-β-gal-positive cells (Figures 5B,C), confirming successful senescence induction. Interestingly, miR-3155b was also downregulated in IR-induced senescent cells, although to a lesser extent than in the DOXO model (Figure 5D).

**FIGURE 5:**
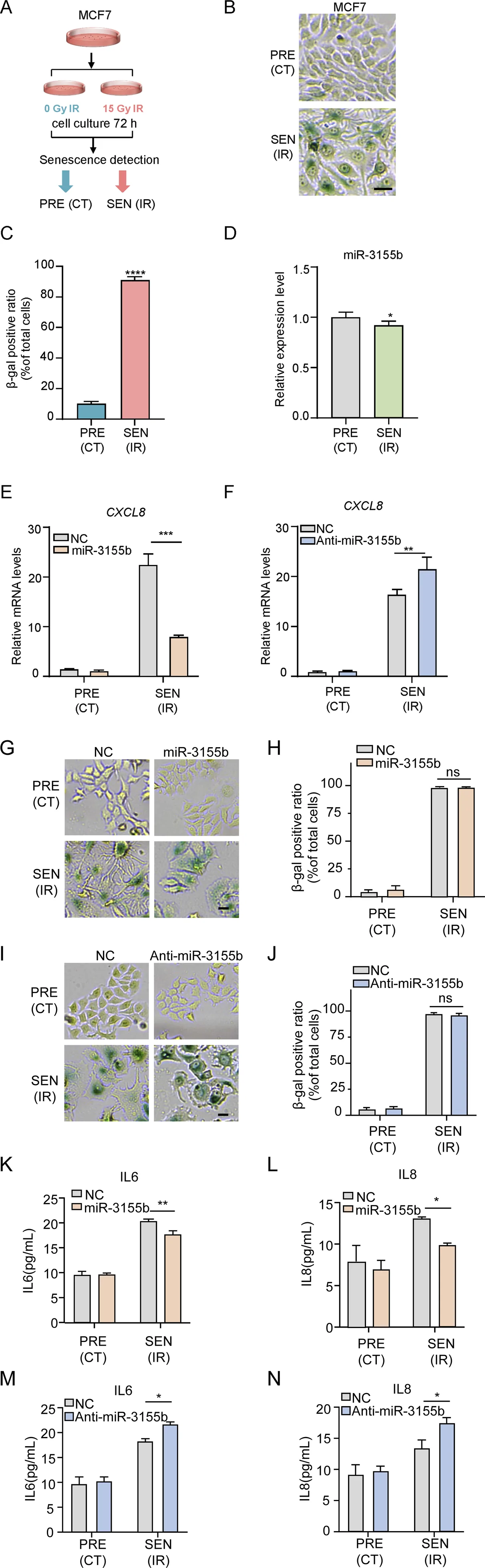
miR-3155b regulates the SASP across distinct TIS models **(A)** Schematic overview of the experimental workflow for X-ray ionizing radiation (IR)-induced senescence in MCF7 cells, comparing presenescent (PRE-CT, 0 Gy control) and senescent (SEN-IR, 15 Gy IR-treated) cells. Cells were further cultured for 3 additional days after IR exposure. **(B,C)** SA-β-gal staining images **(B)** and quantification **(C)** of PRE-CT and SEN-IR cells. Scale bar, 10 μm. **(D)** RT-qPCR analysis of miR-3155b expression in PRE-CT and SEN-IR cells. **(E)** RT-qPCR analysis of CXCL8 mRNA in PRE-CT and SEN-IR cells with or without miR-3155b overexpression. **(F)** RT-qPCR analysis of CXCL8 mRNA in PRE-CT and SEN-IR cells with or without miR-3155b knockdown. **(G,H)** SA-β-gal staining images **(G)** and quantification **(H)** of SEN-IR cells with miR-3155b overexpression or NC. Scale bar, 20 μm. **(I,J)** SA-β-gal staining images **(I)** and quantification **(J)** of SEN-IR cells with miR-3155b knockdown or NC antagomir. Scale bar, 20 μm. **(K, L)** ELISA quantification of secreted IL6 **(K)** and IL8 **(L)** in conditioned medium from SEN-IR cells overexpressing miR-3155b or NC. **(M, N)** ELISA quantification of secreted IL6 **(M)** and IL8 **(N)** in conditioned medium from SEN-IR cells transfected with Anti-miR-3155b or NC antagomir. Data are presented as mean ± SEM from three biological replicates. For B–D, K–N, unpaired two-tailed Student’s t*-*test was used. For E–J, two-way ANOVA with Sidak’s multiple comparisons test was used. *p < 0.05, **p < 0.01, ***p < 0.001 and ****p < 0.0001; ns, not significant.

Subsequently, miR-3155b overexpression and knockdown experiments were performed in IR-induced senescent cells. Overexpression of miR-3155b significantly suppressed the mRNA expression levels of SASP factors, including *IL6*, *CXCL8*, *MMP13*, *TNF*, *IL1B*, and *IFNB1* (Figure 5E; Supplementary Material; Supplementary Figures S4A-E), whereas knockdown of miR-3155b enhanced their expression (Figure 5F; Supplementary Material; Supplementary Figures S5A-E). Importantly, neither overexpression nor knockdown of miR-3155b affected the core senescence program. p21 mRNA and protein levels remained unchanged (Supplementary Material, Supplementary Figures S6A-F), and SA-β-gal positivity was not altered (Figures 5G–J), indicating that the effect of miR-3155b is restricted to the secretory phenotype. ELISA assays further confirmed that miR-3155b overexpression significantly reduced IL6 and IL8 secretion in IR-senescent MCF7 cells (Figures 5K,L). Conversely, miR-3155b knockdown significantly increased the secretion of IL6 and IL8 (Figures 5M,N).

Together, these results indicate that miR-3155b overexpression suppresses the SASP at both the mRNA and protein levels in IR-induced senescence without reversing the senescence state, further supporting its senomorphic function.

### 2.6 Bioinformatic analysis predicts potential targeting of SASP-related mRNAs by miR-3155b

To preliminarily elucidate the molecular mechanism by which miR-3155b regulates SASP factor expression, the miRWalk database was used to predict the targeting relationships between miR-3155b and six SASP factors, including *IL6*, *IL8*, *MMP13*, *TNF*, *IL1B*, and *IFNB1*. miR-3155b was predicted to directly target the mRNA sequences of multiple SASP factors. Specifically, the binding site for *IL8* was located in the 3′untranslated region (3′UTR), whereas binding sites for *IL6*, *TNF*, *IL1B*, and *IFNB1* were located in the coding regions (CDS). No high-confidence binding site was predicted for MMP13 (Table 1).

**TABLE 1:** Summary of miR-3155b predicted targeting modes for SASP factors.

Target	Predicted site	Binding score (max score = 1.0)	Binding energy (kcal/mol)
*CXCL8*	3′UTR	0.923	−19.8
*IL6*	CDS	0.923	−20.3
*TNF*	CDS	0.923	−21.8
*IL1B*	CDS	0.846	−18.5
*IFNB1*	CDS	0.908	−20.3
*MMP13*	None	—	—

RNA duplex structures between miR-3155b and the mRNAs of the predicted targets were modeled using the RNA duplex algorithm as implemented in miRWalk. The predicted base-pairing patterns, including canonical Watson-Crick pairs and G:U wobble pairs, supported the feasibility of direct post-transcriptional repression (Figures 6A–E). Structural elements were color-coded to distinguish paired regions, unpaired single-stranded regions, bulge structures (Figures 6A–E).

**FIGURE 6:**
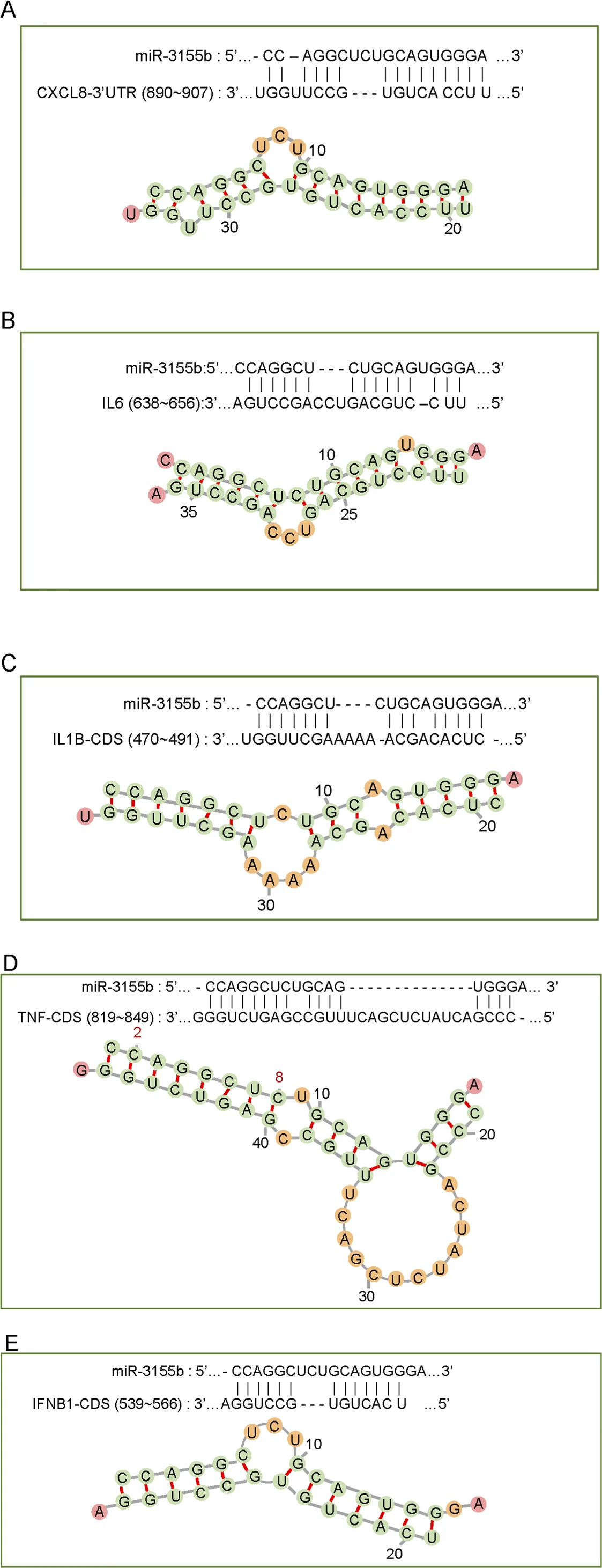
Bioinformatic analysis predicts targeting of SASP-related mRNAs by miR-3155b. **(A–E)** Predicted base-pairing patterns and RNA duplex structures between miR-3155b and the mRNAs of *IL6*, *CXCL8*, *IL1B*, *TNF*, and *IFNB1*, as generated by the RNAduplex algorithm (miRWalk database). In each structure, green indicates paired regions (Watson–Crick or G:U wobble pairs), orange indicates bulge structures, and red indicates unpaired terminal regions.

To assess the reliability of these predictions, we benchmarked the binding scores and binding energy (ΔG) of the predicted duplexes against experimentally validated miR-3155b target sites in miRTarBase (Supplementary Material, Supplementary Figures SA,B; Supplementary Table S1). The binding scores and ΔG values of all five SASP targets fell within the range of experimentally validated sites, confirming that the predicted interactions are thermodynamically favorable.

Collectively, these bioinformatic predictions suggest that miR-3155b may target *CXCL8* 3′UTR and potentially associate with several other SASP-encoding transcripts (*IL6*, *TNF*, *IL1B*, and *IFNB1*), providing a mechanistic basis for the experimental validation described below.

### 2.7 miR-3155b targets *CXCL8* and physically associates with several SASP transcripts

To experimentally validate the predicted interactions between miR-3155b and SASP-encoding mRNAs, we performed luciferase reporter assays. The human *CXCL8* 3′UTR (262–984 bp) and *IFNB1* CDS (293–637 bp) were cloned into the pGLuc-Dura-miR reporter vector. Co-transfection of the *CXCL8* 3′UTR reporter with miR-3155b mimic (100 nM) significantly reduced luciferase activity compared with the negative control (Figure 7A), confirming that miR-3155b targets the *CXCL8* 3′UTR. For the *IFNB1* CDS reporter, miR-3155b mimic showed a tendency to reduce luciferase activity, although this did not reach statistical significance (Figure 7B).

**FIGURE 7:**
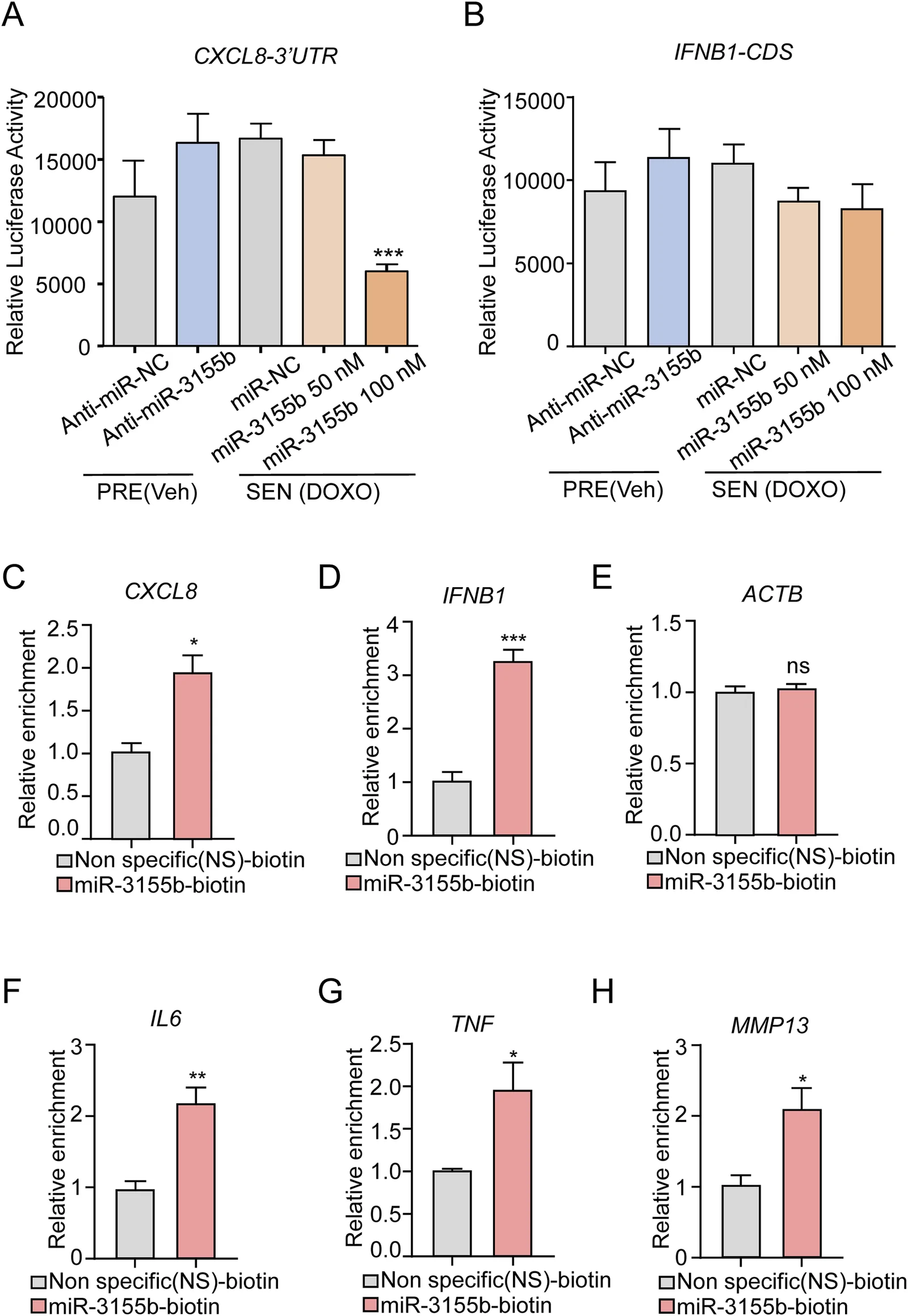
miR-3155b targets *CXCL8* and physically associates with several SASP transcripts **(A,B)** Luciferase reporter assays validating the direct targeting of the *CXCL8* 3′UTR **(A)** and *IFNB1* CDS **(B)** by miR-3155b. The *CXCL8* 3′UTR (262-984 bp) and *IFNB1* CDS (293-637 bp) were cloned into the pGLuc-Dura-miR reporter vector. MCF7 cells were co-transfected with the reporter constructs and miR-3155b mimic (50/100 nM) or NC mimic, or with anti-miR-3155b (50 nM) or NC antagomir, as indicated. **(C–H)** RNA pull-down assay using a 3′-biotinylated miR-3155b probe (miR-3155b-biotin) or a non-specific control probe (NS-biotin) in DOXO-induced senescent MCF7 cells. RT-qPCR analysis of pulled-down *CXCL8* **(C)**, *IFNB1* **(D)**, *ACTB* **(E)**, *IL6* **(F)**, *TNF* **(G)** and *MMP13* **(H)** mRNAs. Data are presented as mean ± SEM from three independent experiments. For A and B, one-way ANOVA was used. For C–H, unpaired two-tailed Student’s t-test was used. *p < 0.05, **p < 0.01 and ***p < 0.001; ns, not significant.

We next performed RNA pull-down assays using a 3′-biotinylated miR-3155b probe to capture miRNA–mRNA complexes in DOXO-induced senescent MCF7 cells. Both *CXCL8* and *IFNB1* mRNAs were significantly enriched in the miR-3155b pull-down fraction compared with the non-specific control probe (Figures 7C,D). As a negative control, *ACTB* mRNA was not enriched (Figure 7E).

Importantly, the other predicted CDS-targeted SASP factors, *IL6* (Figure 7F) and *TNF* (Figure 7G), were also significantly enriched by the miR-3155b probe. Notably, *MMP13* mRNA, which lacks a predicted miR-3155b binding site, was enriched in the pull-down fraction (Figure 7H), suggesting that miR-3155b may physically associate with *MMP13* through previously unrecognized binding sites or via indirect protein-mediated interactions.

Collectively, these data demonstrate that miR-3155b targets the *CXCL8* 3′UTR and physically associates with *IFNB1*, *IL6*, *TNF*, and MMP13.

### 2.8 miR-3155b senomorphic function extends to T47D cells

To assess whether the above findings extend to other luminal breast cancer cells, we examined miR-3155b function in T47D cells. Treatment with increasing concentrations of DOXO (0, 30, and 60 nM) for 72 h induced a dose-dependent increase in SA-β-gal-positive cells, with 60 nM producing the most robust response (Supplementary Material, Supplementary Figures S8A,B), accompanied by dose-dependent upregulation of *CDKN1A*, *IL6*, and *CXCL8* mRNA (Supplementary Material, Supplementary Figures S8C-E). Therefore, 60 nM DOXO was selected as the senescence-inducing condition for subsequent experiments.

In senescent T47D cells, miR-3155b was similarly downregulated (Supplementary Material, Supplementary Figure S8F), consistent with the MCF7 results. Overexpression of miR-3155b did not affect *CDKN1A* mRNA levels (Supplementary Material, Supplementary Figure S8GF), but significantly suppressed *IL6* and *CXCL8* mRNA expression (Supplementary Material, Supplementary Figures S8H,I). Conversely, knockdown of miR-3155b upregulated *IL6* and *CXCL8* mRNA expression (Supplementary Material, Supplementary Figures S8J,K). ELISA assays further confirmed that miR-3155b overexpression significantly reduced the secretion of IL6 and IL8 proteins (Supplementary Material, Supplementary Figures S8L,M).

Collectively, these results demonstrate that miR-3155b suppresses SASP factor expression and secretion without affecting the senescence state in T47D cells, indicating that its SASP-regulatory function extends to at least two Luminal A breast cancer cell lines.

## 3 Discussion

### 3.1 Screening and senomorphic function of miR-3155b

In this study, we employed the Luminal A breast cancer cell line MCF7 as the primary model, together with doxorubicin (DOXO) and ionizing radiation (IR) as two clinically relevant modalities, to investigate the role of miR-3155b in therapy-induced senescence (TIS). Small RNA sequencing identified multiple differentially expressed miRNAs, yet only miR-3155b was consistently confirmed by RT-qPCR in both models. Among the five non-validated candidates, hsa-miR-1269a, hsa-miR-509-3-5p, hsa-miR-514a-3p, and hsa-miR-10523-5p exhibited low read counts (Readcount_TPM <10), suggesting that their differential signals likely arose from noise near the detection limit; hsa-miR-122b-3p displayed double melting peaks in qPCR analysis, indicative of poor primer specificity. Notably, all of these failed candidates met the same statistical thresholds (|log_2_FC| > 1 and *q* < 0.05) used to select miR-3155b, underscoring the limitation of relying solely on statistical significance to distinguish genuine biological signals from sequencing noise. In contrast, miR-3155b not only withstood independent validation but also exhibited higher read counts and a larger effect size (log_2_FC < −2), supporting its selection as a reliable candidate for further investigation.

Building on this screening, we found that miR-3155b was downregulated in TIS. Its overexpression significantly suppressed the mRNA levels of *IL6*, *CXCL8*, *MMP13*, *TNF*, *IL1B*, and *IFNB1*, and ELISA assays further demonstrated that the secreted protein levels of IL6 and IL8 were also reduced. Conversely, knockdown of miR-3155b increased both the mRNA levels of these SASP factors and the secretion of IL6 and IL8. Importantly, neither overexpression nor knockdown of miR-3155b significantly altered SA-β-gal activity, Ki67 expression, γH2AX and 53BP1 foci formation, or the mRNA and protein levels of p21. These effects were observed in both DOXO- and IR-induced TIS models. In the T47D Luminal A cell line, we confirmed the downregulation of miR-3155b upon DOXO-induced senescence, the suppression of *IL6* and *CXCL8* mRNA by miR-3155b overexpression, the upregulation of these factors by miR-3155b knockdown, and the lack of effect on *p21* mRNA. Therefore, our conclusions are presently most applicable to Luminal A breast cancer.

### 3.2 Mechanistic basis for the targeting of SASP factors by miR-3155b

At the mechanistic level, luciferase reporter assays confirmed that *CXCL8* is targeted by miR-3155b through its canonical 3′UTR. For *IFNB1*, miR-3155b mimic showed a tendency to reduce the luciferase activity of the CDS reporter, although this did not reach statistical significance. miRNAs targeting 3′UTRs typically recruit the RNA-induced silencing complex (RISC) to promote efficient mRNA degradation, resulting in robust suppression (Li et al., 2018; Shen et al., 2020; Qu et al., 2015; Tangtanatakul et al., 2017; Hynes and Kakumani, 2024; Bartel, 2009; Huntzinger and Izaurralde, 2011). In contrast, the predicted binding sites for *IL6*, *TNF*, *IL1B*, and *IFNB1* reside in coding sequences (CDS) (Bartel, 2009; Huntzinger and Izaurralde, 2011; Sapkota et al., 2023), where miRNA binding predominantly mediates translational repression with weaker effects on mRNA stability due to competition with translating ribosomes (Hausser et al., 2013). This mechanistic distinction between 3′UTR- and CDS-mediated regulation may explain the more pronounced mRNA reduction observed for *CXCL8* relative to *IL6*. ELISA data showed an approximately 50% reduction in secreted IL6 protein, suggesting that modest changes at the mRNA level may be amplified at the protein level. IL6 is a potent cytokine that functions in a paracrine manner at picomolar concentrations; partial reductions in its secretion have been shown to exert meaningful biological effects in the tumor microenvironment, including attenuated STAT3 activation, reduced recruitment of myeloid-derived suppressor cells, and altered angiogenesis (Zhong et al., 2016; Farhana et al., 2024).

Using *in vitro* RNA pull-down with a biotinylated miR-3155b probe and *CXCL8* as a positive control and *ACTB* as a negative control, we detected significant enrichment of *IFNB1*, *IL6*, and *TNF* mRNAs, suggesting that miR-3155b may physically associate with these transcripts. Additionally, MMP13 lacks a predicted miR-3155b binding site, yet its mRNA was also enriched in the RNA pull-down assay, suggesting that miR-3155b may physically associate with *MMP13* mRNA through previously unrecognized binding sites or via indirect protein-mediated interactions. Beyond post-transcriptional regulation, miRNAs have also been reported to interact with target gene promoters to modulate transcription (Sarkar and Kumar, 2025), and the overall intricacy of miRNA–target interactions may be underestimated (Diener et al., 2024); such mechanisms could possibly involve upstream transcription factors such as NF-κB or STAT3 (Han et al., 2020; Wang et al., 2024), adding another potential layer to the regulatory complexity. The precise mode of *MMP13* regulation awaits further investigation.

It should be noted that the predicted duplex structure of *TNF* contains an internal gap of approximately 10–11 nucleotides, which is structurally atypical compared with canonical miRNA–target pairing. Although RNA pull-down assays detected enrichment of *TNF* mRNA, this observation does not address whether this non-canonical gap affects RISC engagement geometry or downstream repression. Therefore, *TNF* should currently be regarded as a candidate physical associate of miR-3155b, and whether this predicted site directly mediates the observed regulation awaits validation by luciferase reporter or mutational analysis. These structural features are also consistent with the broader complexity of miRNA–target interactions beyond canonical models.

### 3.3 Methodological considerations and model comparison

Several methodological aspects warrant careful consideration. Regarding overexpression levels, a substantial fraction of the initial ∼32,000-fold apparent overexpression was attributable to extracellular mimic contamination. After additional PBS washes to remove residual mimic, the overexpression level was revised to approximately 15,000-fold, which nonetheless remains above the physiological range. Only the 20 nM mimic condition produced detectable SASP suppression, whereas lower concentrations (2 nM and 0.2 nM) did not. This dose–response pattern raises two possibilities: the SASP-suppressive effect genuinely requires supraphysiological levels of miR-3155b, or a degree of non-specific silencing contributes at high mimic concentrations. To evaluate the latter, we examined five control transcripts unrelated to SASP or senescence—*GAPDH*, *ACTB*, *RPL13A*, *TBP*, and *PGK1*—and observed no significant changes at the 20 nM overexpression condition. These results do not suggest widespread off-target effects. Nevertheless, the current system cannot entirely exclude the potential contribution of non-specific effects. Future studies employing CRISPR-mediated miR-3155b knockout or overexpression driven by an endogenous promoter will be needed to validate its regulatory function at physiological expression levels.

In comparing the two senescence models, the downregulation of miR-3155b was less pronounced in IR-induced senescence than in DOXO-induced senescence (approximately 0.85 vs. 0.5 relative to control), which may reflect differences in the kinetics of senescence progression (Kim et al., 2023). A distinction should be drawn between two scenarios: exogenous delivery of miR-3155b can still suppress the aforementioned SASP factors in IR-senescent cells, indicating that the downstream regulatory machinery remains intact across stimuli; however, as an endogenous regulator, miR-3155b likely plays a more prominent role in DOXO-induced senescence. Hence, the IR model primarily provides supportive evidence that this regulatory axis can be exogenously activated.

### 3.4 Study limitations and future directions

Several limitations of this study should be considered. First, protein-level validation was restricted to IL6 and IL8, with bidirectional confirmation limited to these two factors; the secreted protein levels of MMP13, TNF, IL1B, and IFNB1 were not assessed by ELISA. Second, the T47D validation included only IL6 and CXCL8 and did not comprehensively verify senomorphic specificity. Third, CDKN2A promoter hypermethylation leads to p16 silencing in MCF7 and T47D cells (Ozdemir et al., 2025). We confirmed the absence of *p16* mRNA in MCF7 cells by RT-qPCR, using HeLa and MEF cells as positive controls, but this inherent limitation of the model means that our assessment of senescence stability relies primarily on the combined evidence from p21 and SA-β-gal. Fourth, in the IR-induced senescence model, the endogenous downregulation of miR-3155b was considerably smaller than that in the DOXO model, suggesting a more limited role as an endogenous SASP regulator in the IR context. Thus, the IR model mainly serves as supporting evidence that this regulatory axis can be exogenously engaged, rather than establishing an equally important endogenous function in IR-induced senescence. Fifth, our validation was performed only in two Luminal A cell lines; extension of these findings to Luminal B and other breast cancer subtypes awaits future investigation.

Regarding mechanisms that remain to be validated, the atypical structural gap in the predicted TNF duplex and its binding mode require further elucidation, and the targeting relationship of IL1B also awaits experimental confirmation. The mode of physical association between MMP13 and miR-3155b, direct non-canonical binding or protein-bridged interaction, remains to be clarified, as does the potential involvement of upstream transcription factors such as NF-κB or STAT3 (Han et al., 2020; Wang et al., 2024), which could be tested by ChIP or pathway inhibitor experiments. Furthermore, the current RNA pull-down experiments did not include a specificity control (e.g., RNase competition or a scrambled/seed-mutant probe). Therefore, non-specific signal or protein-mediated indirect interactions cannot be excluded at this stage. Future studies incorporating such controls, together with luciferase reporter assays, will be needed to further clarify the binding status of these candidate targets.

Whether the pronounced suppression of IFNB1 involves the cGAS-STING pathway (Billimoria and Bhatt, 2025) remains an intriguing hypothesis: two predicted miR-3155b binding sites in cGAS mRNA are supported by published CLIP-seq data (miRTarBase entry MIRT540177), but CLIP-seq evidence reflects physical protein–RNA association, which differs in strength from functional validation by reporter assays. Therefore, this hypothesis awaits further functional testing. Finally, whether miR-3155b functions as an endogenous SASP regulator under physiological expression levels can be addressed in future studies using CRISPR-mediated knockout or endogenous promoter-driven overexpression.

From a therapeutic perspective, this study aligns with the emerging senomorphic strategy, which aims to suppress the deleterious effects of the SASP without eliminating senescent cells (Mchugh et al., 2025; Zhong et al., 2016; Farhana et al., 2024; Sarkar and Kumar, 2025; Diener et al., 2024; Han et al., 2020; Wang et al., 2024; Kim et al., 2023; Dong et al., 2024; Saliev and Singh, 2025; Cuollo et al., 2020; Jiang et al., 2025). As an endogenous non-coding RNA, miR-3155b offers advantages such as low immunogenicity and compatibility with nucleic acid delivery platforms, making it a promising candidate for SASP-directed therapy in Luminal A breast cancer.

In summary, this study identifies miR-3155b as a senomorphic microRNA in the context of TIS in Luminal A breast cancer. miR-3155b can regulate the expression and secretion of IL6, CXCL8, MMP13, TNF, IL1B, and IFNB1 without overtly altering the senescence phenotype, with consistent effects observed across two TIS models and two Luminal A cell lines. These findings advance our understanding of miRNA-mediated SASP regulation in TIS and nominate miR-3155b as a potential molecular target for mitigating the pro-tumorigenic consequences of the SASP in Luminal A breast cancer.

## 4 Materials and methods

### 4.1 Cell culture and transfection

MCF7 cells were purchased from the American Type Culture Collection (ATCC, RRID: CVCL_0031) and maintained in Dulbecco’s modified Eagle medium (DMEM; Corning, 10-013-CVRC) supplemented with 10% fetal bovine serum (10% FBS; Excel, FND500). T47D cells were purchased from medical research center, Sun Yat-sen Memorial Hospital, and cultured in RPMI1640 supplemented with 10% FBS. Both cell lines were incubated at 37 °C in a humidified atmosphere containing 5% CO_2_. For transfection, cells were seeded in 6-well plates 24 h prior to transfection. When cell confluence reached 60%–70%, transfection was performed using RNAi Pro Transfection Reagent (MIKX, mk4018) in accordance with the manufacturer’s instructions. miRNA-3155b mimics/antagomirs and the corresponding negative control were synthesized by Tsingke Biotech Co., Ltd. (Beijing, China) and used at a final concentration of 20 nM. Cells were harvested 48 h post-transfection.

### 4.2 Therapy-induced senescence models

Doxorubicin treatment: MCF7 cells were treated with 120 nM doxorubicin (Santa Cruz, sc-200923) for 72 h to induce senescence. T47D cells were treated with 60 nM doxorubicin for 72 h to induce senescence. Control cells were treated with an equal volume of dimethyl sulfoxide (DMSO; Sigma, 276,855) as vehicle.

Ionizing radiation treatment: MCF7 cells were exposed to 15 Gy of X-ray irradiation and further cultured for 3 additional days. Control cells were maintained under the same conditions without irradiation.

### 4.3 Cell proliferation analysis

Cell proliferation was assessed using the Cell Counting Kit-8 (CCK-8, Dojindo, CK04). MCF7 cells were seeded into 96-well plates at a density of 750 cells per well, with five replicates per group. On the following day, the medium was replaced with 100 μL of fresh medium containing 10% CCK-8 reagent, and the cells were incubated at 37 °C for 1 h. Subsequently, 90 μL of the supernatant was transferred to a new plate, and the absorbance was measured at 450 nm. This procedure was repeated daily. Population doublings (PDs) were calculated from the absorbance readings, and growth curves were generated by plotting PDs against culture days.

### 4.4 Quantitative real-time PCR

Total RNA was extracted from cells using TRIzol reagent (TaKaRa, 9109), followed by cDNA synthesis with the PrimeScript RT Reagent Kit (Takara, RR047A). Quantitative real-time PCR (qPCR) was conducted using TB Green® Premix Ex Taq™ II (TaKaRa, RR820B), with β-Actin serving as the reference gene. For miRNA detection, reverse transcription was performed using stem-loop primers, with U6 small nuclear RNA (U6 snRNA) as the internal control.

Primer sequences are as follows. β-Actin: 5′-AGA GCT ACG AGC TGC CTG AC-3′ (F), 5′-AGC ACT GTG TTG GCG TAC AG-3′ (R). IL6: 5′-ACT CAC CTC TTC AGA ACG AAT TG-3′ (F), 5′-CCA​TCT​TTG​GAA​GGT​TCA​GGT​TG-3′ (R). IL8: 5′-ACT GAG AGT GAT TGA GAG TGG AC-3′ (F), 5′-AAC CCT CTG CAC CCA GTT TTC-3′ (R). MMP13: 5′-TCG CGT CAT GCC AGC AAA TTC CAT-3′ (F), 5′-TTC TTC CCC TAC CCC GCA CTT CTG-3′ (R). TNF(TNF-α)*:* 5′-CTC TTC TGC CTG CAC TTT G-3′ (F), 5′-ATG GGC TAC AGG CTT GTC ACT C-3′ (R). IL1B: 5′-ATG ATG GCT TAT TAC AGT GGC AA-3′ (F), 5′- GTC GGA GAT TCG TAG CTG GA-3′ (R). IFNB1: 5′-GCC ATC AGT CAC TTA AAC AGC-3′ (F), 5′-GAA ACT GAA GAT CTC CTA GCC T-3′ (R). p21: 5′-TCC AGC GAC CTT CCT CAT CCA C-3′ (F), 5′-TCC ATA GCC TCT ACT GCC ACC ATC-3′ (R). U6 snRNA: 5′-TGC GGT GCT CGC TTC GGC AGC-3′ (F), 5′-CCA GTG CAG GGT CCG AGT-3′ (R). miR-3155b: 5′-CGC GCC AGG CTC TGC A-3′ (F), 5′-AGT GCA GGG TCC GAG GTA TT-3′ (R).

### 4.5 Senescence-associated β-Galactosidase (SA-β-gal) staining

Cells in 6-well plates were fixed with β-gal staining fixative (Beyotime, C0602) for 10 min at room temperature, washed three times with phosphate-buffered saline (PBS), and then incubated with staining solution containing X-gal. The plates were sealed and incubated at 37 °C without CO_2_ overnight. SA-β-gal-positive cells were observed under a light microscope, and the percentage of senescent cells was calculated.

### 4.6 Immunofluorescence

Cells cultured on coverslips were fixed with 4% paraformaldehyde for 20 min at room temperature, permeabilized with 0.5% Triton X-100 for 10 min, and blocked with 5% bovine serum albumin (BSA, Solarbio, A8020) for 30 min. Samples were incubated overnight at 4 °C with primary antibodies against γH2AX (Millipore Cat# 05-636, RRID:AB_309864), Ki67 (Cell Signaling Technology Cat# 9129, RRID:AB_2687446), or 53BP1 (Novus Cat# NB100-304, RRID:AB_10003037). After washing with PBS, cells were incubated with fluorescence conjugated secondary antibodies (Thermo Fisher Scientific Cat# A-11001, RRID:AB_2534069 and Cat# A-31570, RRID:AB_2536180)) for 1 h at room temperature in the dark. Nuclei were counterstained with DAPI (Vector Laboratories Cat# H-1200, RRID:AB_2336790). Images were acquired using a confocal microscope, and fluorescence intensity was analyzed with ImageJ software (Version 1.54p, RRID: SCR_003070).

### 4.7 Western blot

Cells were washed with PBS and lysed on ice for 10 min in RIPA buffer containing 1% protease inhibitor cocktail. Lysates were sonicated briefly and centrifuged at 12,000 × *g* for 10 min at 4 °C. Protein concentrations were determined by BCA assay. Equal amounts of protein were denatured in loading buffer at 100 °C for 5 min, separated by SDS-PAGE, and transferred to nitrocellulose membranes using a semi-dry transfer system (0.3 A, 1 h). Membranes were blocked with 3% bovine serum albumin (BSA, Solarbio, A8020) in TBST for 30 min and incubated overnight at 4 °C with primary antibodies against p21 (Calbiochem Cat# OP64, RRID:AB_213274) or GAPDH (Abmart Cat# P30008M). After washing with TBST, membranes were incubated with IRDye 680RD (LI-COR Cat# 926-68070, RRID:AB_10956566) or IRDye 800CW (LI-COR Cat# 926-32211, RRID:AB_621843) secondary antibodies for 1 h at room temperature in the dark. Signals were detected using an Odyssey imaging system (LI-COR).

### 4.8 Enzyme-linked immunosorbent assay (ELISA)

IL6 and IL8 levels in cell culture supernatants were measured using human IL6 (J&L Biological, Cat# JL14113-48T) and IL8 (J&L Biological, Cat# JL19291-48T) double-sandwich biotin-avidin ELISA kits. Supernatants were centrifuged at 1,000 × *g* for 20 min at 4 °C to remove debris and stored at −80 °C until analysis. Assay plates were incubated with 100 μL of standards or samples for 60 min at 37 °C, followed by sequential incubation with biotinylated detection antibody (60 min) and HRP-conjugate (30 min) with washing steps between. TMB substrate was added for 15 min in the dark, and the reaction was terminated with stop solution. Absorbance was measured at 450 nm, and cytokine concentrations were calculated from standard curves.

### 4.9 Luciferase assay

3′UTR of Human CXCL8 mRNA (262-984 bp) was cloned into pGLuc-Dura-miR plasmids to generate pGLuc-IL8 UTR luciferase reporter system. CDS of human IFNB1 mRNA (293-637 bp) was cloned into mutated pGLuc-Dura-miR vector which erased TAA stop codon to generate pGLuc-IFNB1 CDS luciferase reporter system. MCF7 cells were seeded in 24-well plates at a density of 1 × 10^5^ cells/well with or without DOXO treatment (120 nM) and cultured to the second day to reach 60%–70% confluence before transfection. Cells were co-transfected with pGLuc-IL8 UTR or pGLuc-IFNB1 CDS and miR-3155b/NC antagomir or miR-3155b mimic/NC using RNAi Pro (MIKX, mk4018) transfection reagent. Co-transfection groups included pGLuc vector (500 ng per well), miR-3155b/NC antagomir (50 nM per well) or miR-3155b mimic/NC (100 nM for miR-NC and 50/100 nM for miR-3155b mimic per well). The transfection medium was replaced with fresh complete medium 4–6 h post-transfection. After 48 h of continuous culture with/without DOXO treatment, the cell supernatants were collected and sequentially detected using Gaussia Luciferase Assay Kit (Beyotime, China) in 96 microplates using Synergy GTX multi-mode reader (BioTek). All experiments were performed in three independent biological replicates.

### 4.10 RNA Pulldown assay

Biotin-labeled miRNA pull-down was performed to assess the binding of miR-3155 to target mRNAs. Briefly, two 6-cm dishes of DOXO-induced senescent MCF7 cells were harvested and lysed on ice for 15 min in 200 μL of ice-cold lysis buffer containing 20 mM Tris-HCl pH 7.4, 100 mM NaCl, 0.5 mM EDTA, 0.5% NP-40, protease inhibitors, and RNase inhibitor. After centrifugation at 12,000 *g* for 10 min at 4 °C, 10 μL of the cleared lysate was saved as input. For each pull-down, 2 μg of a 3′-biotinylated miR-3155 probe or a 3′-biotinylated non-specific control probe (NS-biotin) was diluted in 10 μL DEPC water, denatured at 95 °C for 3 min, chilled on ice for 2 min, and then added to the remaining lysate together with additional RNase inhibitor. The mixture was incubated with rotation at 4 °C for 1 h, followed by the addition of 20 μL streptavidin-conjugated magnetic beads (Thermo, 88,817), and incubation for another 30 min. Beads were washed 3× with wash buffer, and RNA from both input and pull-down samples was isolated using TRIzol reagent. After reverse transcription, target mRNA such as IL6 was quantified by qPCR. Enrichment relative to the non-specific control probe (NS-biotin) was determined by the ΔΔCt method: ΔCt = Ct (IP) – Ct (Input), ΔΔCt = ΔCt (miR-3155) – ΔCt (NS-biotin), fold enrichment = 2^−ΔΔCt^.

### 4.11 small RNA sequencing

MCF7 cells from the DMSO young control (PRE-Veh) and doxorubicin-induced senescent (SEN-DOXO) groups were collected, with three biological replicates per group. Cells were lysed using TRIzol reagent and submitted to Novogene Bioinformatics Technology Co., Ltd. (Beijing, China) for small RNA high-throughput sequencing.

For qualified small RNA samples, 3′ and 5′ adapters were ligated to the corresponding ends of RNA fragments, respectively. First-strand cDNA was synthesized by reverse transcription and further amplified by PCR to generate double-stranded cDNA libraries. The libraries were purified and size-selected to obtain fragments with an insert length of 18–40 bp. After quality control using a Qubit Fluorometer, real-time quantitative PCR, and an Agilent 2100 Bioanalyzer, qualified libraries were sequenced on an Illumina platform in single-end 50-bp (SE50) mode.

Raw sequencing data in fastq format were processed using custom Perl and Python scripts. Low-quality reads, reads containing poly-N, reads with 5′ adapter contamination, and reads lacking 3′ adapters or insert tags were removed to obtain clean reads. Q20, Q30, and GC content of the clean reads were calculated for quality assessment. Clean reads were mapped to the human reference genome without mismatches using Bowtie software (Langmead et al., 2009).

Known miRNAs were identified by aligning mapped small RNA tags against miRBase (v20) (Kozomara and Griffiths-Jones, 2014) using miRDeep2 (Friedländer et al., 2012), and secondary structures of potential miRNAs were analyzed. To remove non-miRNA tags, small RNA tags were aligned to the RepeatMasker and Rfam databases to filter out non-miRNA sequences.

miRNA expression levels were normalized and quantified as transcripts per million (TPM) (Zhou et al., 2010). Differential expression analysis between PRE(Veh) and SEN (DOXO) groups was performed using the DESeq R package (anders and Huber, 2010), and miRNAs with |log_2_FC (fold changes)| > 1 and adjusted *p* value (*q* value) < 0.05 were considered significantly differentially expressed (Storey, 2003).

Pearson correlation analysis was used to evaluate the reproducibility of biological replicates within each group. Data sorting and processing were performed using Microsoft Excel, and volcano plots were generated using GraphPad Prism 8.0 to visualize the distribution of significantly differentially expressed miRNAs.

### 4.12 Bioinformatic analysis

Prediction of miR-3155b target genes was performed using the miRWalk database (http://mirwalk.umm.uni-heidelberg.de/, RRID:SCR_016509), with intersections from miRDB database (https://mirdb.org/, RRID:SCR_010848) and miRTarBase (mirtarbase.cuhk.edu.cn/, RRID:SCR_017355). Gene Ontology (GO) enrichment analysis was conducted using Metascape (http://metascape.org/, RRID:SCR_016620), with a significance threshold of *p* value < 0.05. Base pairing patterns between miR-3155b and the mRNAs of the target genes, along with RNA duplex structure predictions, were derived from the miRWalk database.

### 4.13 Gene Ontology enrichment analysis

GO enrichment analysis was performed using Metascape (http://metascape.org) with *Homo sapiens* as the background. A total of 532 predicted target genes of miR-3155b were obtained from miRWalk using a stringent cutoff of Seed = 1 (Supplementary Table S2). The following ontology categories were analyzed: GO Molecular Functions, GO Biological Processes, and KEGG Pathway. Significance was determined using a threshold of *p* value <0.01, with a minimum overlap of 2 genes and a minimum enrichment factor of 1.5. A total of 401 significantly enriched terms were identified, and the complete list is provided in Supplementary Table S3.

### 4.14 Statistical analysis

All experiments were conducted with three independent biological replicates. Data are expressed as mean ± standard error of the mean (SEM). Comparisons between two groups were evaluated using unpaired two-tailed Student’s t-test. For multiple group comparisons, two-way ANOVA followed by Sidak’s multiple comparisons test was applied, with significance indicated by *p < 0.05, **p < 0.01, ***p < 0.001, ****p < 0.0001, and not significant (ns). Statistical analyses were performed using GraphPad Prism 8.0 (RRID: SCR_002798), and *p* < 0.05 was considered statistically significant.

## Data Availability

The original contributions presented in the study are included in the article/Supplementary Material, further inquiries can be directed to the corresponding authors.
