# Supplementary material for: Senomorphic miR-3155b suppresses the senescence-associated secretory phenotype in therapy-induced senescence
Source: Front Cell Dev Biol. 2026 Sep 28;14:1868137. doi: 10.3389/fcell.2026.1868137 (PMC13655142; doi:10.3389/fcell.2026.1868137)

Supplementary Figure 1

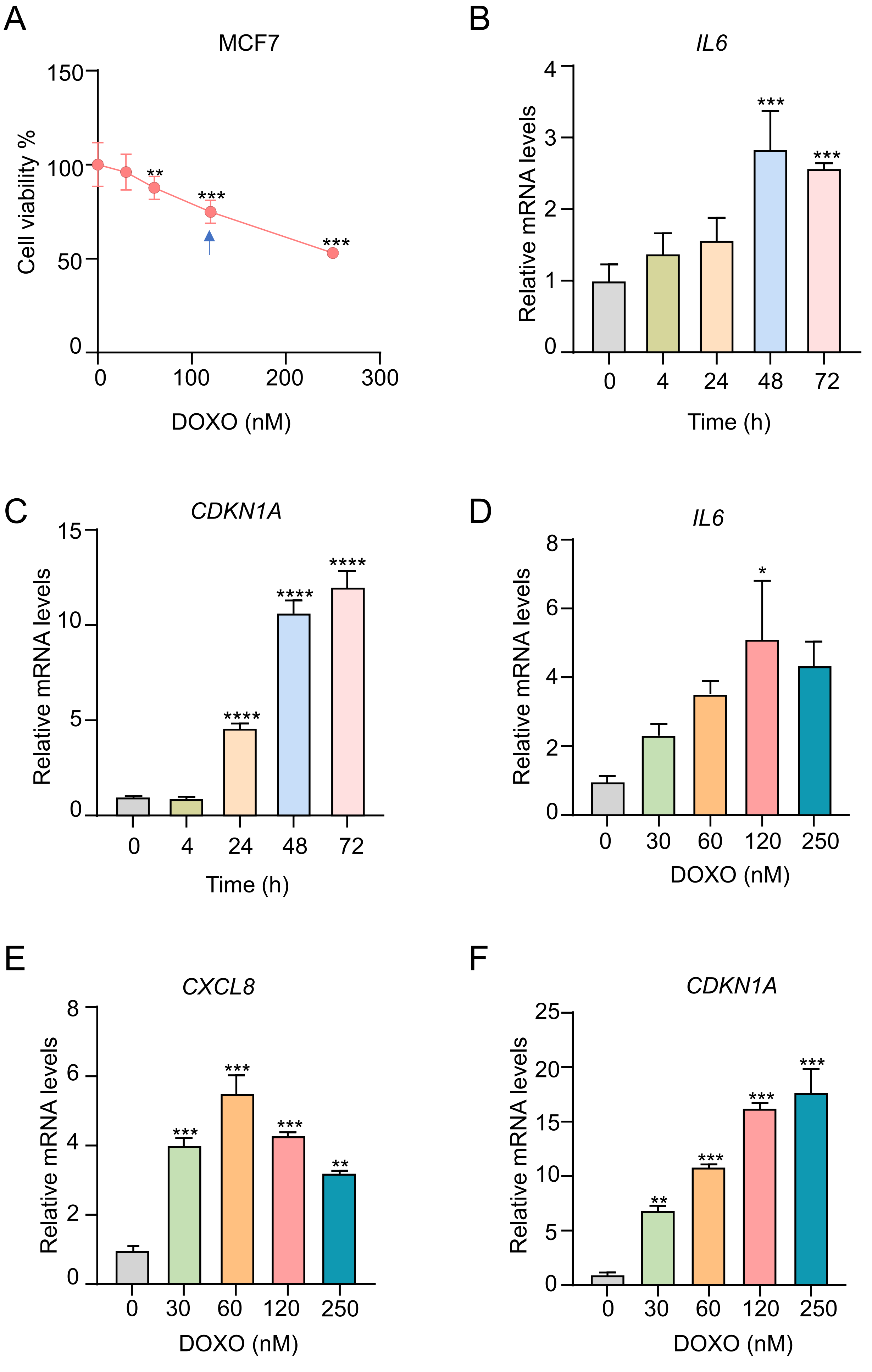

Supplementary Figure 2

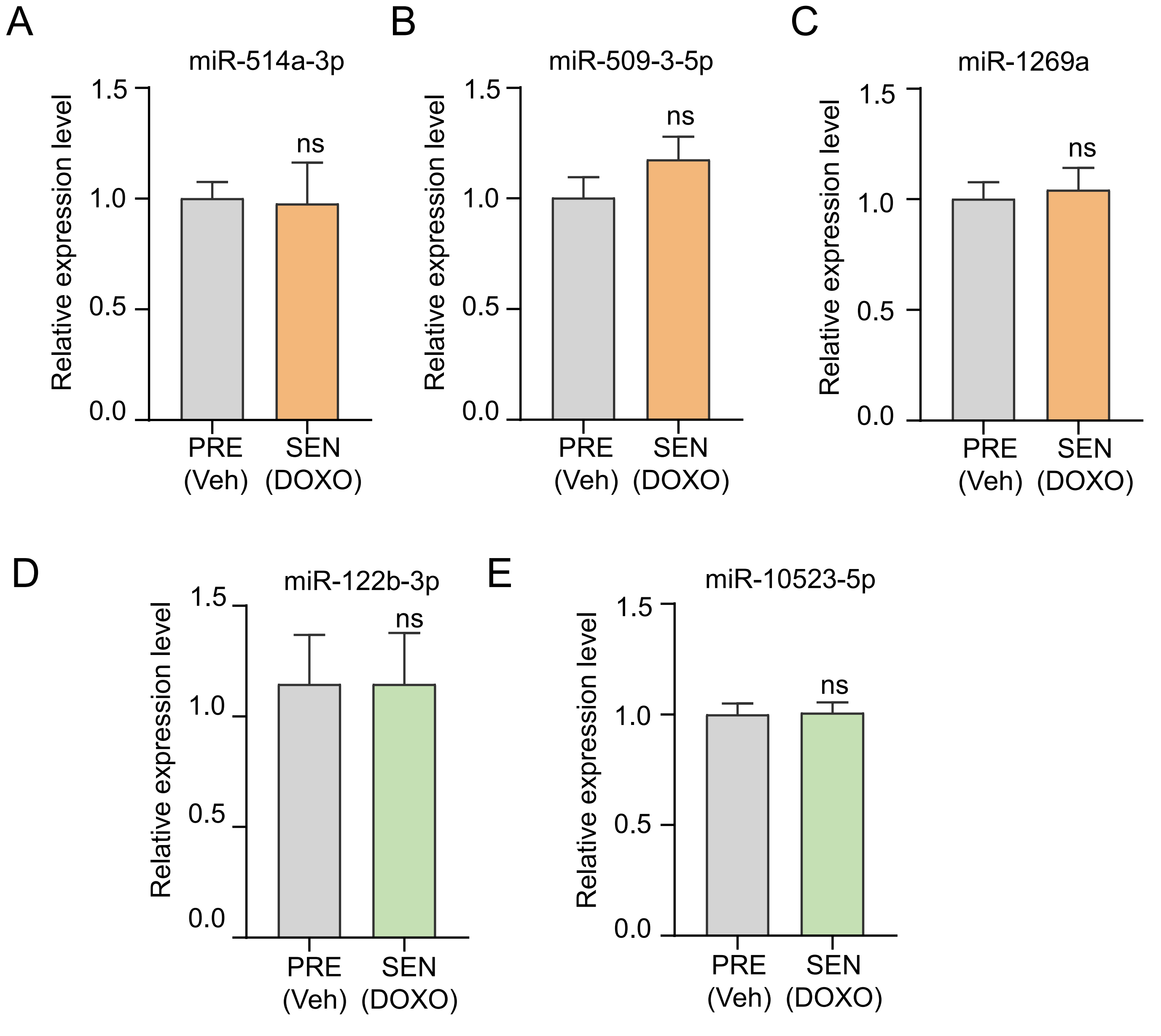

Supplementary Figure 3

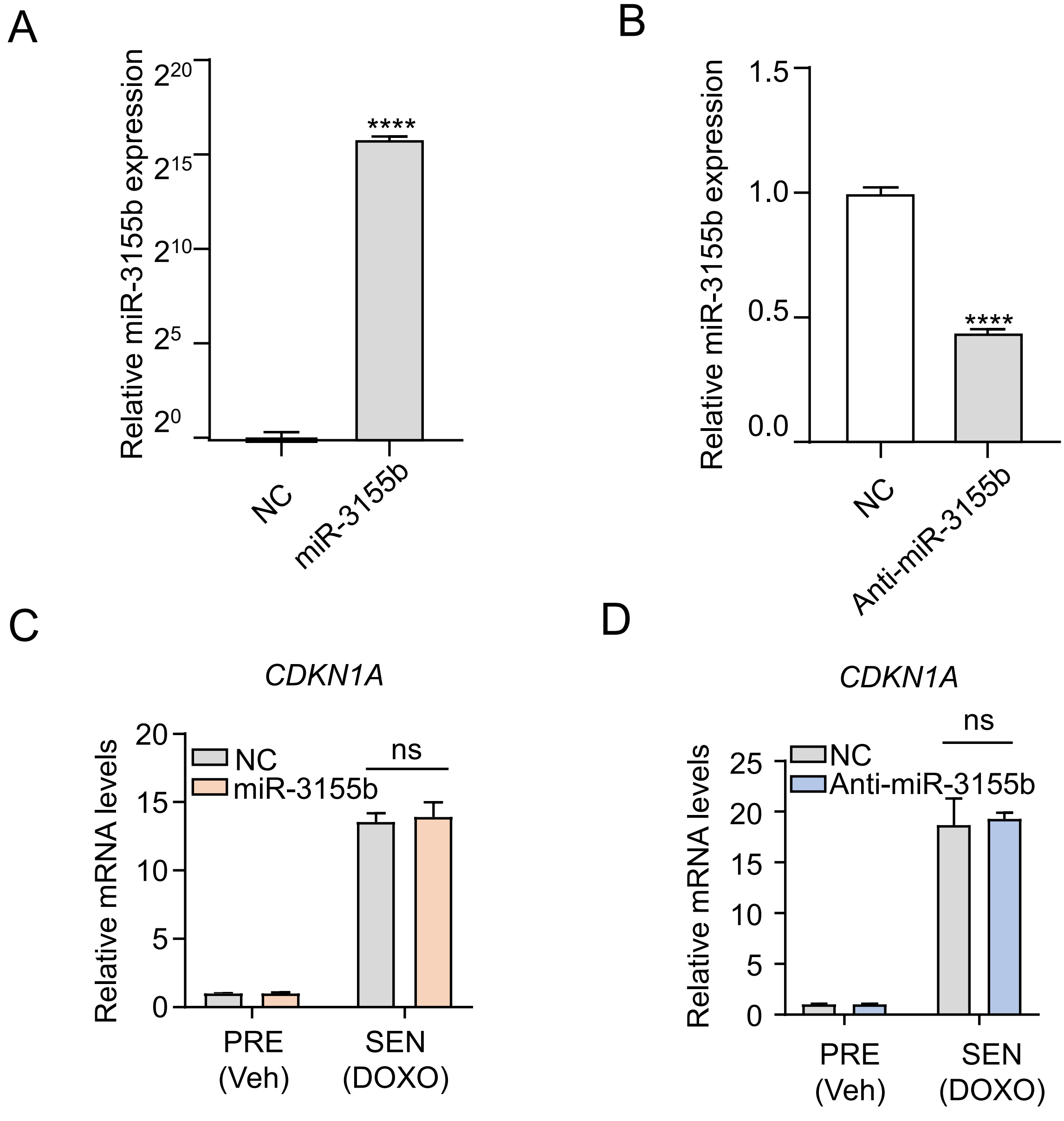

Supplementary Figure 3

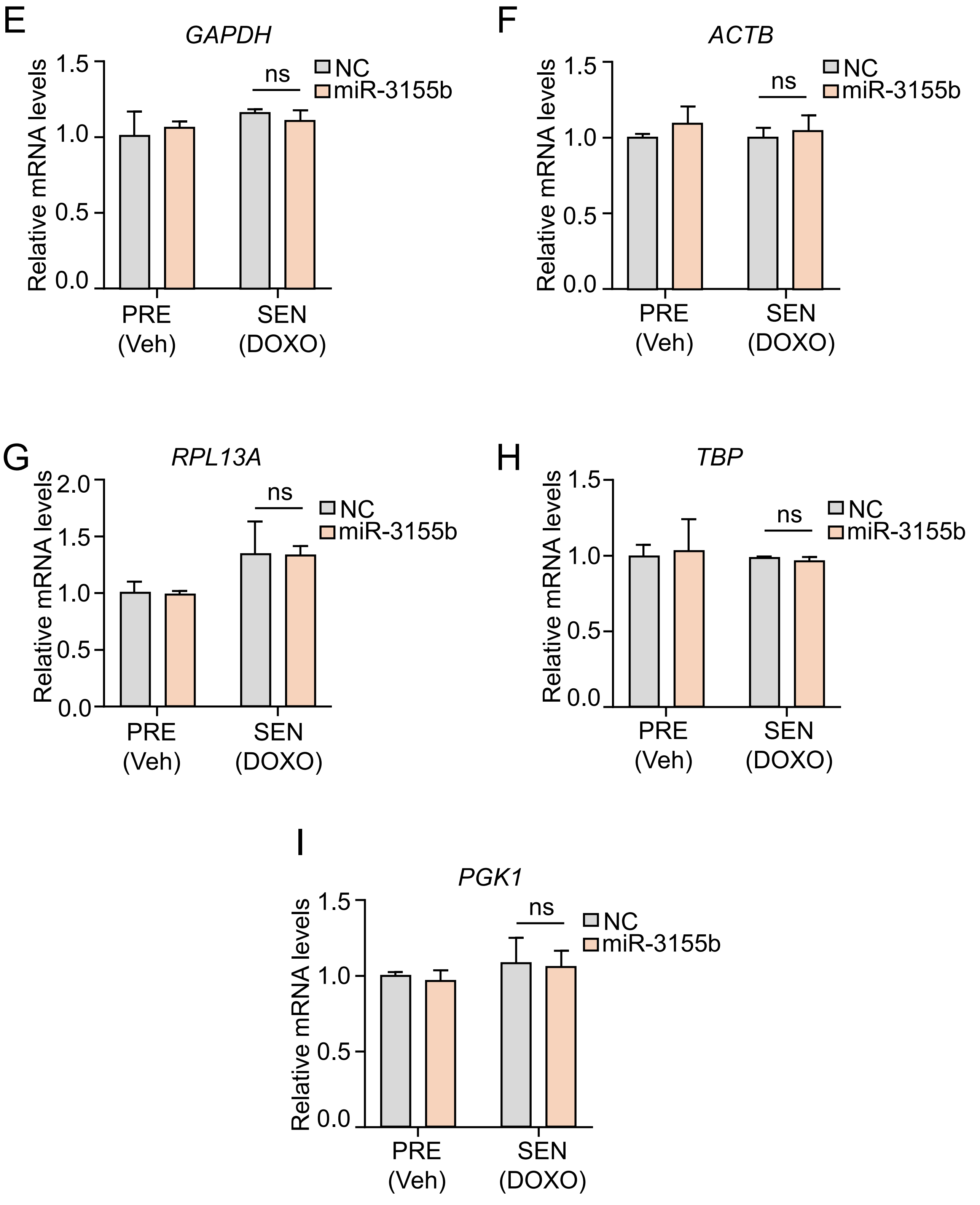

Supplementary Figure 4

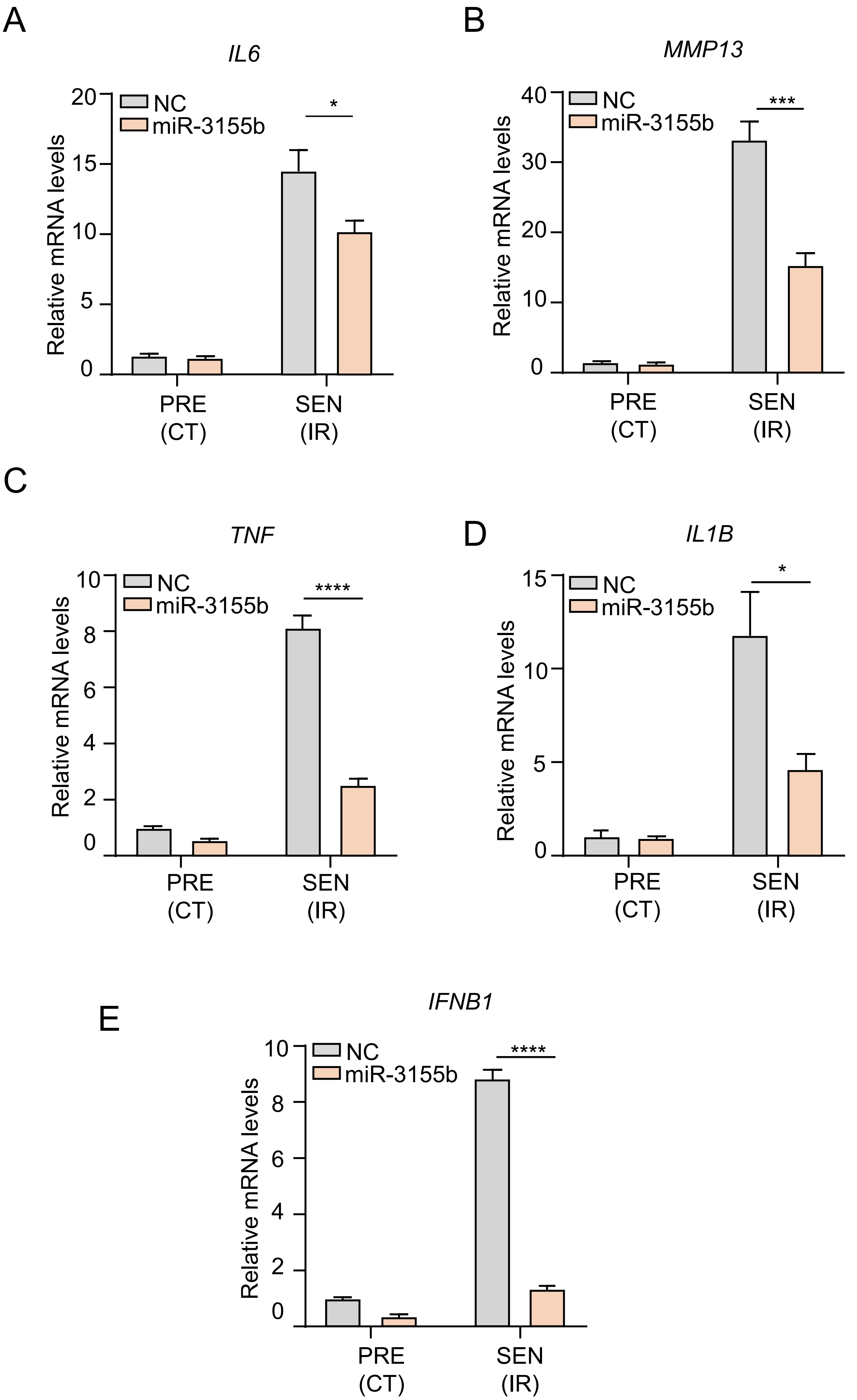

Supplementary Figure 5

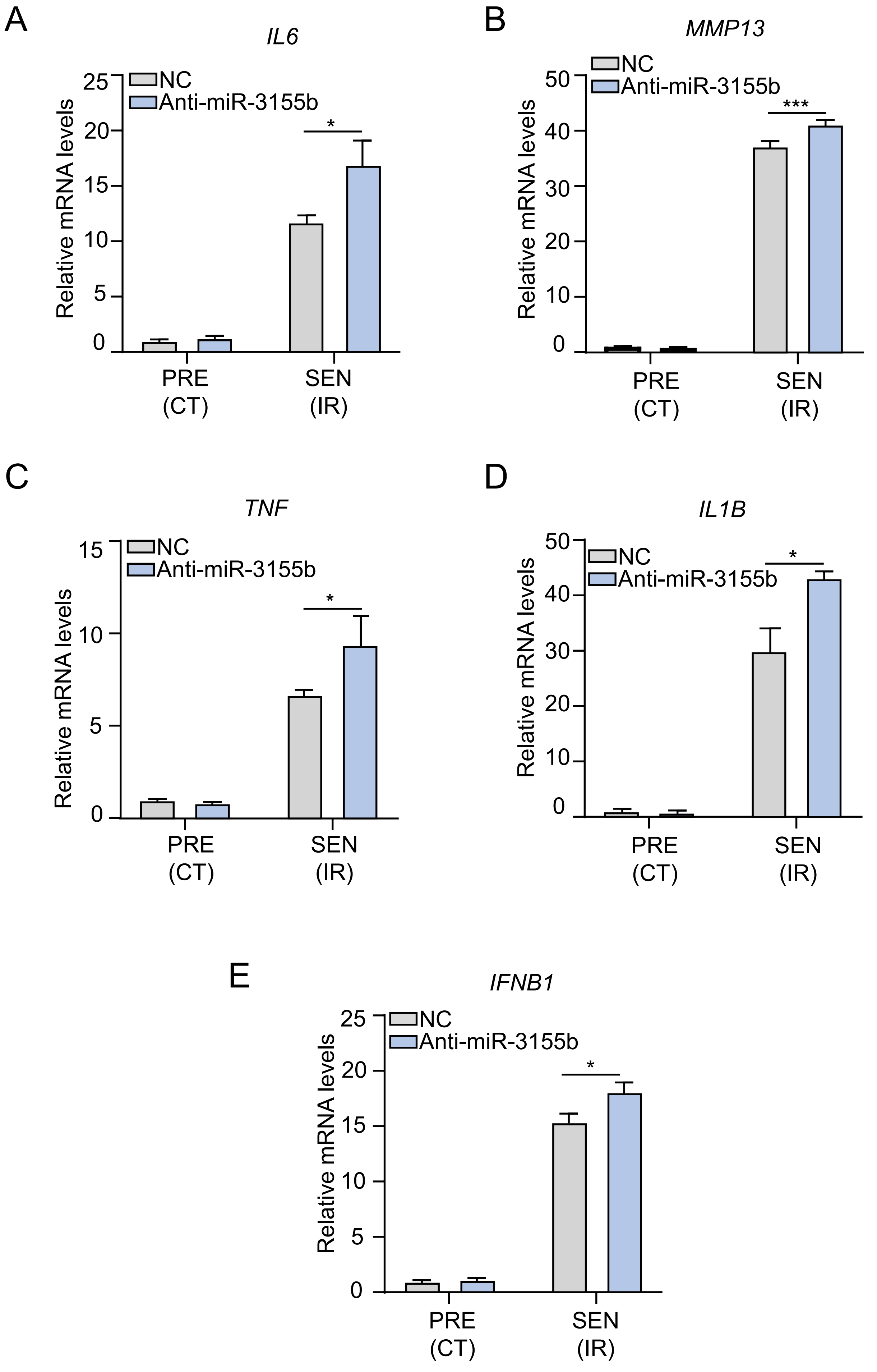

Supplementary Figure 6

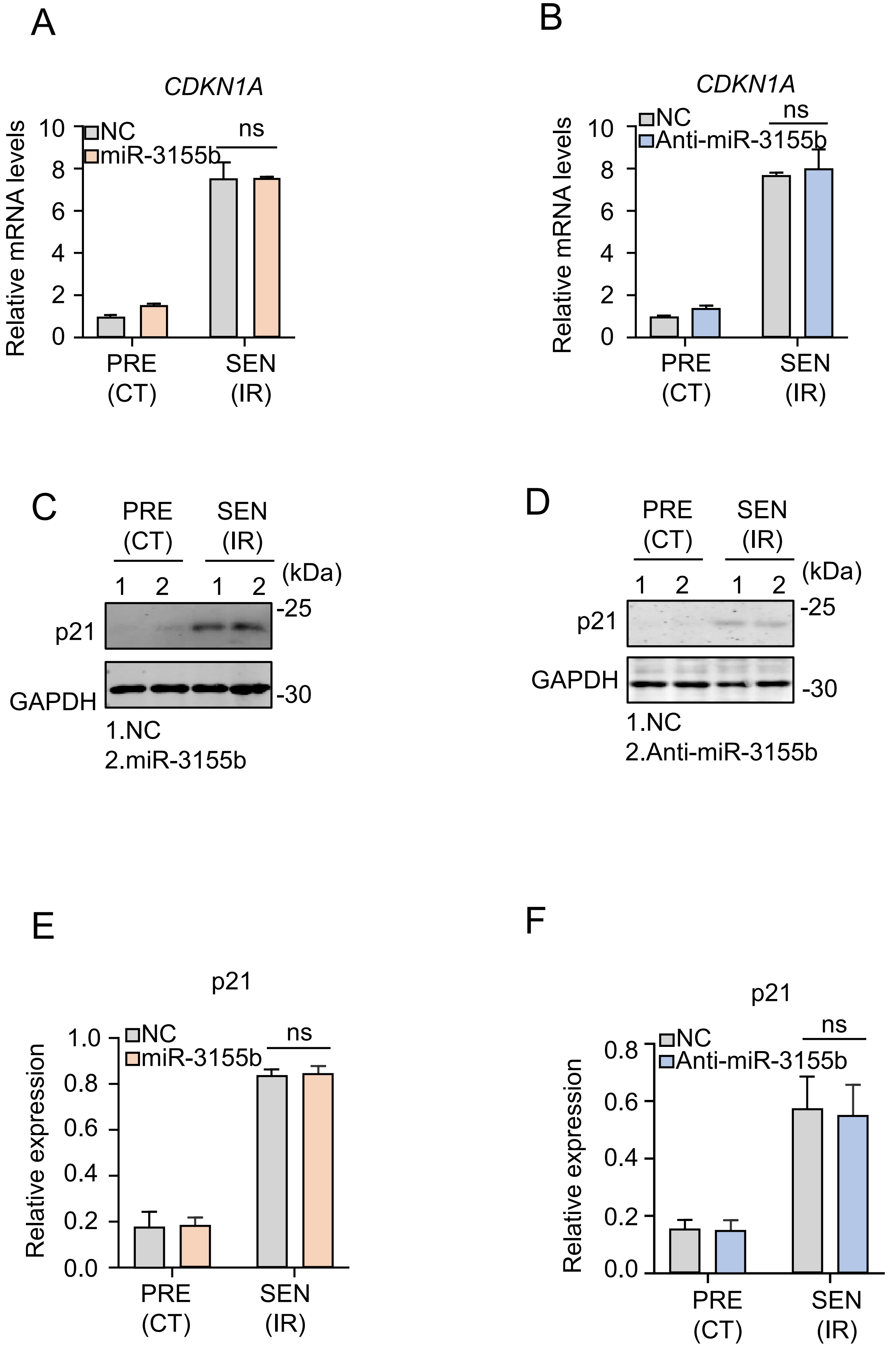

Supplementary Figure 7

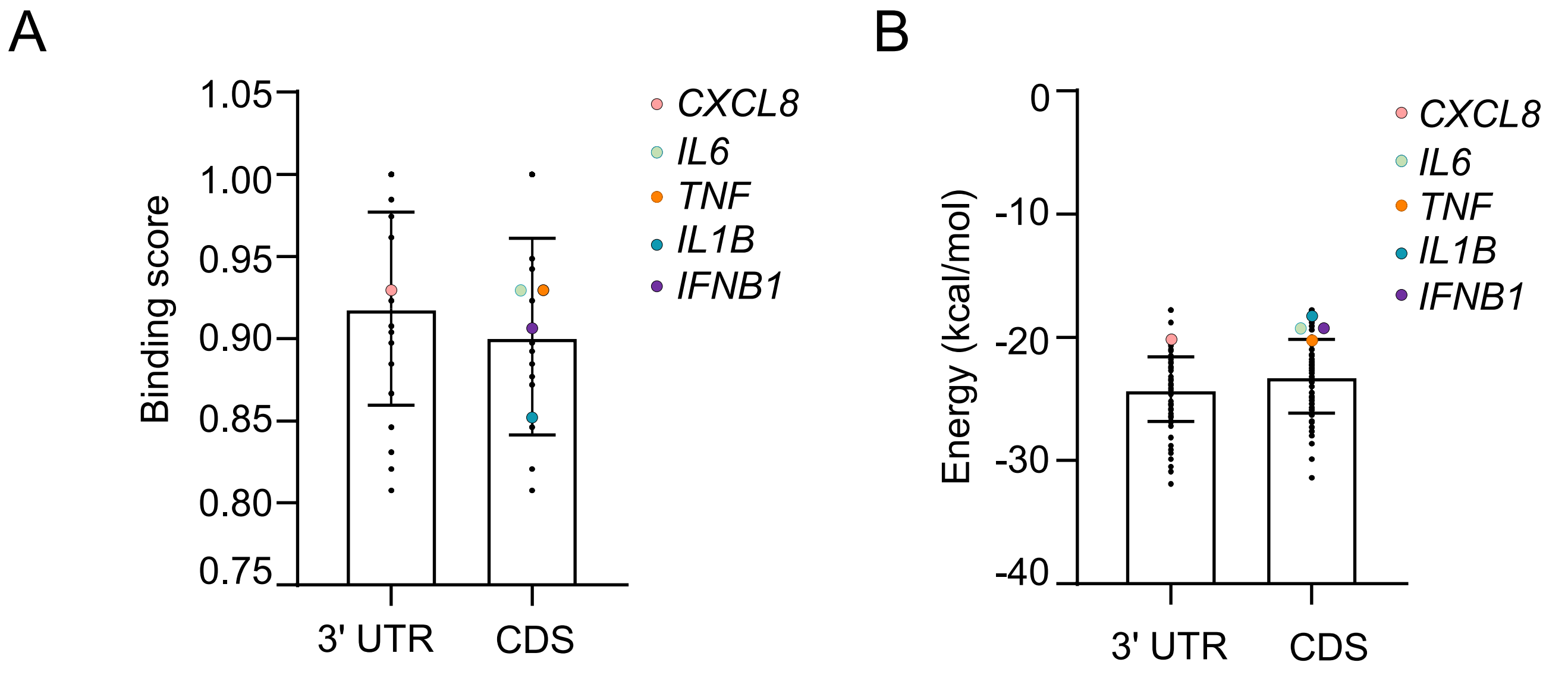

Supplementary Figure 8

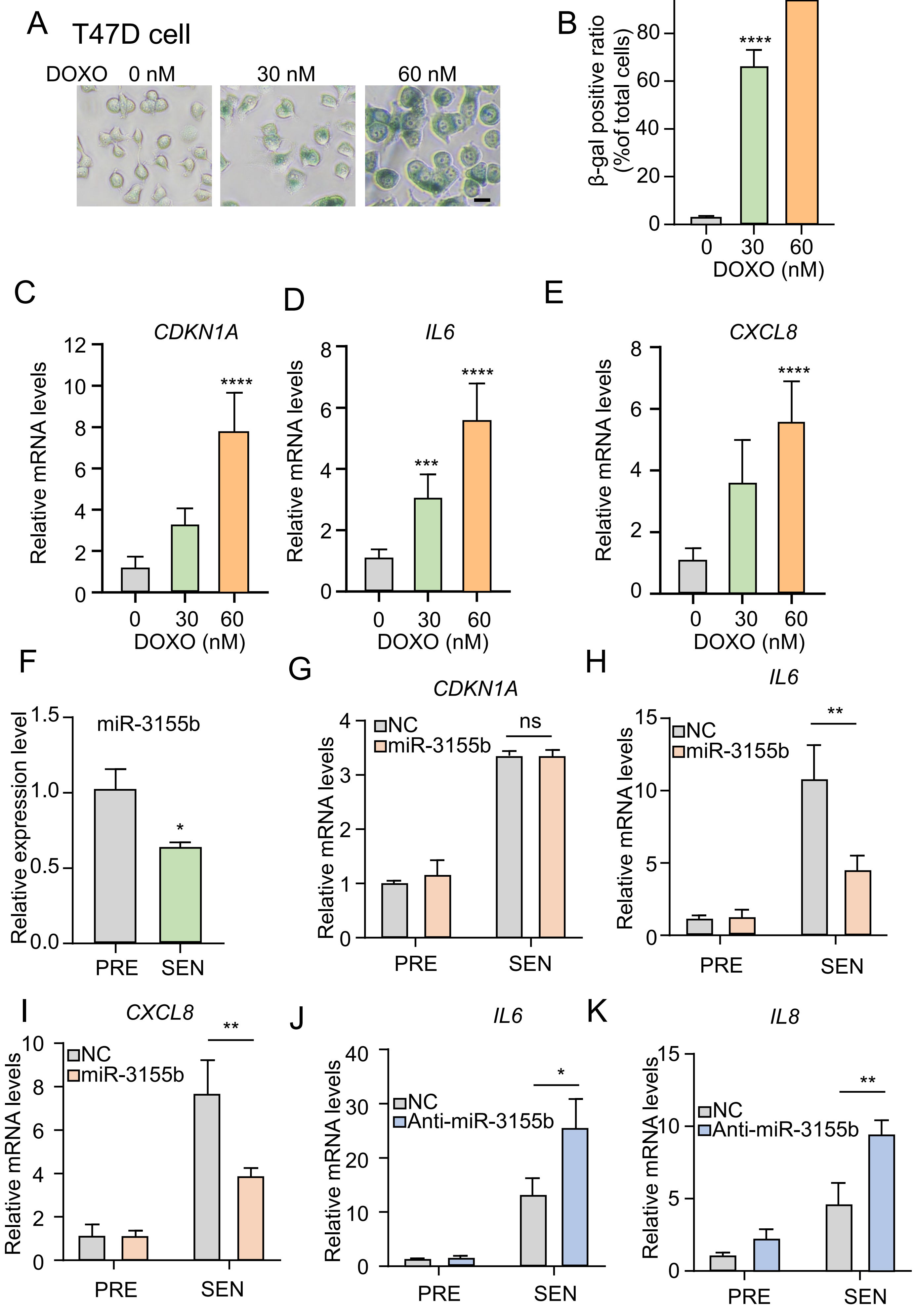

Supplementary Figure 8

L

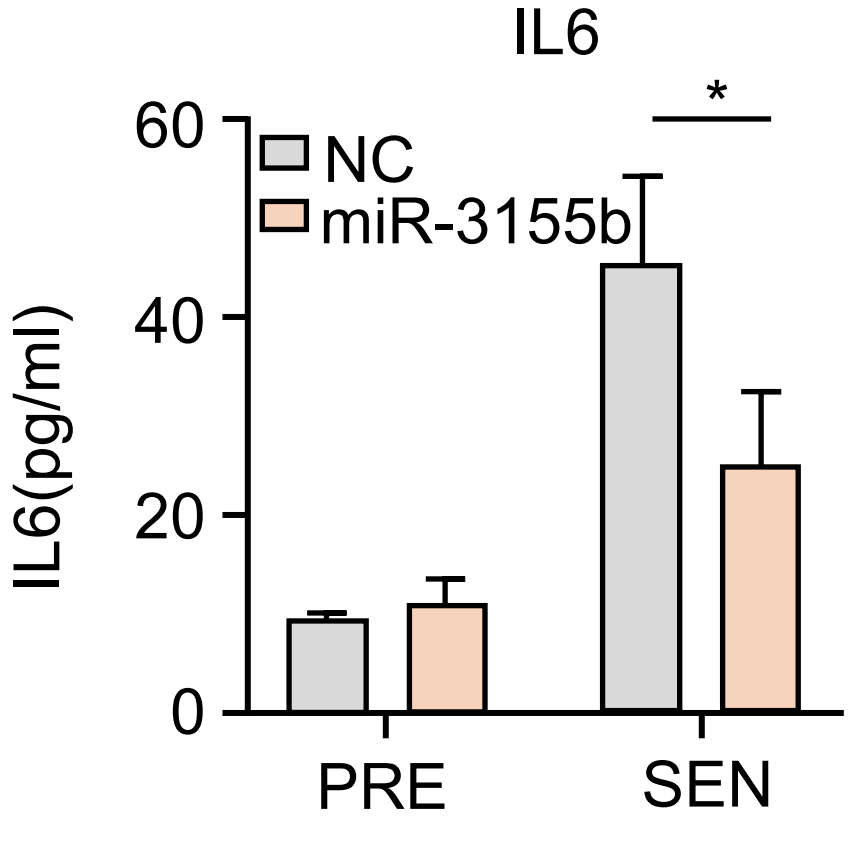

M

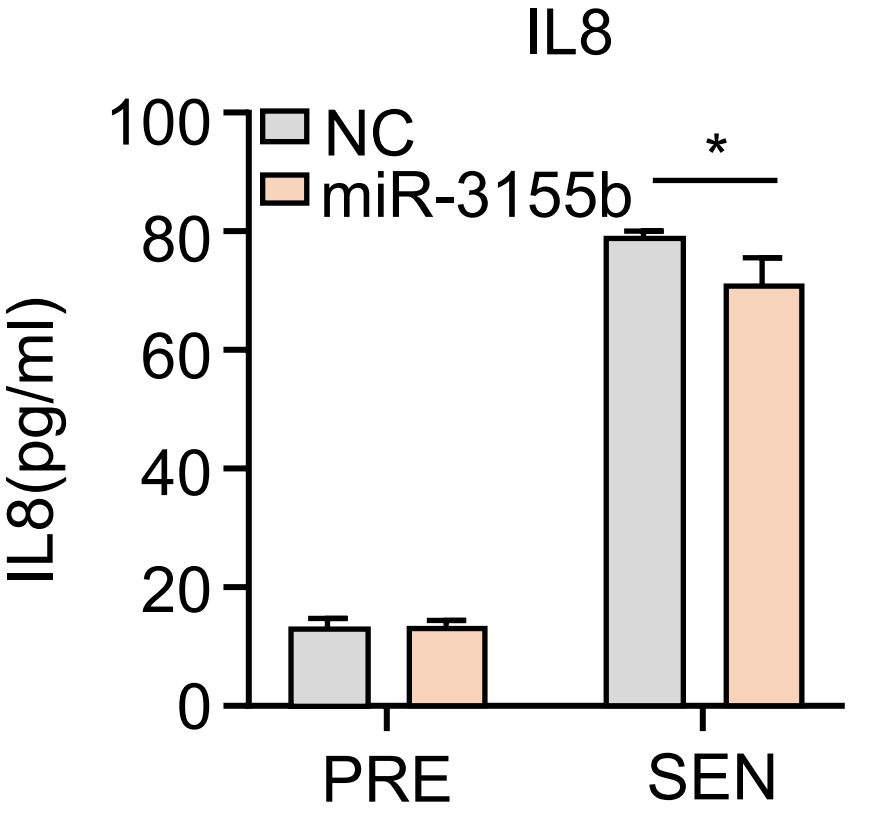

Supplement: Supplementary file 1 [file DataSheet1.pdf]
